# CDK Substrate Phosphorylation and Ordering the Cell Cycle

**DOI:** 10.1016/j.cell.2016.11.034

**Published:** 2016-12-15

**Authors:** Matthew P. Swaffer, Andrew W. Jones, Helen R. Flynn, Ambrosius P. Snijders, Paul Nurse

**Affiliations:** 1 Cell Cycle Laboratory, The Francis Crick Institute, London NW1 1AT, UK; 2 Protein Analysis and Proteomics Platform, The Francis Crick Institute, London NW1 1AT, UK; 3 Laboratory of Yeast Genetics and Cell Biology, Rockefeller University, New York, NY 10065, USA

**Keywords:** CDK, cyclin-dependent kinase, cell cycle, S phase, mitosis, phosphorylation, kinase, phosphoproteomics

## Abstract

S phase and mitotic onset are brought about by the action of multiple different cyclin-CDK complexes. However, it has been suggested that changes in the total level of CDK kinase activity, rather than substrate specificity, drive the temporal ordering of S phase and mitosis. Here, we present a phosphoproteomics-based systems analysis of CDK substrates in fission yeast and demonstrate that the phosphorylation of different CDK substrates can be temporally ordered during the cell cycle by a single cyclin-CDK. This is achieved by rising CDK activity and the differential sensitivity of substrates to CDK activity over a wide dynamic range. This is combined with rapid phosphorylation turnover to generate clearly resolved substrate-specific activity thresholds, which in turn ensures the appropriate ordering of downstream cell-cycle events. Comparative analysis with wild-type cells expressing multiple cyclin-CDK complexes reveals how cyclin-substrate specificity works alongside activity thresholds to fine-tune the patterns of substrate phosphorylation.

## Highlights

• Global analysis of CDK substrates and their phosphorylation dynamics in fission yeast
• CDK substrate phosphorylation can be temporally ordered by a single cyclin-CDK
• Substrate-specific activity thresholds set phosphorylation timing and cell-cycle order
• Cyclin-substrate specificity provides a secondary, more minor, level of regulation

## Graphical Abstract

**Figure:**
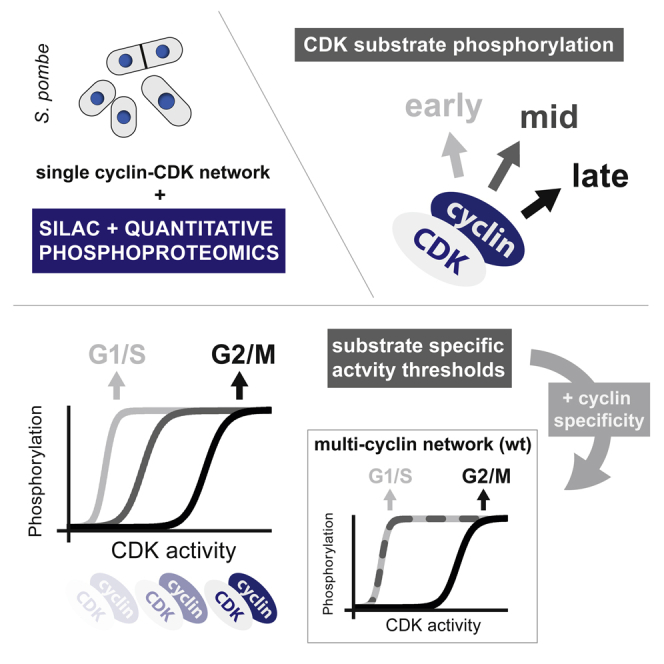

## Teaser

A systems analysis of CDK substrates in yeast shows that the phosphorylation of different CDK substrates can be temporally ordered during the cell cycle by a single cyclin-CDK via rising CDK activity and the differential sensitivity of substrates to CDK activity.

## Introduction

The proper timing and alternation of DNA replication (S phase) and chromosome segregation (mitosis) is essential for the faithful propagation of the eukaryotic genome and the production of viable daughter cells. Progression from G1 into S phase and from G2 into mitosis is controlled by the protein kinase activities of multiple different cyclin-CDK (cyclin dependent kinase) complexes, which are differentially expressed during the cell cycle. The biochemical characterization of budding yeast and metazoa cyclin-CDK complexes has illustrated that different cyclin-CDK complexes can exhibit distinct specificity toward subsets of substrates (Bhaduri and Pryciak, 2011, Brown et al., 1999, Kõivomägi et al., 2011, Loog and Morgan, 2005, Pagliuca et al., 2011, Peeper et al., 1993). This has led to the generally accepted view that the temporal ordering of S phase and mitosis is achieved by the biochemical specificity of different cyclin-CDKs, resulting in different subsets of critical substrates becoming phosphorylated as different cyclin-CDK complexes are expressed.

A difficulty with this model is that specific cyclin-CDKs can be eliminated in a range of eukaryotes without majorly impacting cell-cycle order (reviewed in Uhlmann et al., 2011). This includes the viable genetic deletion of cyclins and CDKs in murine cell lines (Geng et al., 2003, Kalaszczynska et al., 2009, Kozar et al., 2004, Santamaría et al., 2007) and the demonstration that a mitotic cyclin can initiate S phase in *Xenopus* egg extracts (Moore et al., 2003). This apparent plasticity suggests that the substrate specificity of different cyclin-CDKs may be less important than is generally appreciated. The most extreme example of such plasticity is in the fission yeast *Schizosaccharomyces pombe*, where a single genetically engineered cyclin-CDK chimera between the mitotic B-type cyclin Cdc13 and the CDK Cdc2 (Cdc13-L-Cdc2) can substitute for the four cyclin-CDK complexes acting during the mitotic cell cycle and the six cyclin-CDK complexes acting during the meiotic cell cycle (Coudreuse and Nurse, 2010, Gutiérrez-Escribano and Nurse, 2015). This has led to an alternative hypothesis, which states that quantitative changes in the level of CDK activity, rather than specificity, are critical for the orderly initiation of S phase and mitosis (Stern and Nurse, 1996). Support for this model has come from the demonstration in *S. pombe* that different concentrations of a CDK inhibitor block DNA replication and chromosome segregation, suggesting that a lower CDK activity threshold may be required for S phase than mitosis (Coudreuse and Nurse, 2010). However, current evidence for this hypothesis has been limited to genetic or physiological observations, while biochemical studies have focused on cyclin specificity. As such, there is a lack of molecular information about the phosphorylation of CDK substrates with respect to cell-cycle temporal order and the changes in in vivo CDK activity during the cell cycle, both of which are necessary to adequately evaluate the activity threshold model.

Here, we present an in vivo systems analysis of CDK substrate phosphorylation to directly examine this. Experimentally addressing this problem in vivo is confounded by the complexity of the cell-cycle control network. Influenced by synthetic biology thinking, we have used the genetically engineered simplification of this network in *S. pombe* (*cdc13-L-cdc2 cdc2Δ cdc13Δ cig1Δ cig2Δ puc1Δ*) (Coudreuse and Nurse, 2010) to overcome the difficulties encountered when comparing activity thresholds if several different cyclin-CDKs are present. We have combined this synthetic approach with a SILAC (stable isotope labeling with amino acids in cell culture; Ong et al., 2002) and phosphoproteomics-based systems analysis to rigorously identify hundreds of CDK substrates and quantify their phosphorylation dynamics. As part of this, we have developed a phosphoproteomics-based methodology to measure changes in protein kinase activity in vivo. Our data provide direct evidence that a monotonic rise in activity of a single cyclin-CDK can temporally order substrate phosphorylation by passage through sequential substrate-specific activity thresholds. We also show that the differential sensitivity of substrates to CDK activity is likely to be established, at least in part, by substrate affinity for cyclin-CDK. These observations and previous genetic studies have only been able to consider what *can* happen as opposed to what *does* happen because, by necessity, they involve the removal of certain factors in the network (Coudreuse and Nurse, 2010, Fisher and Nurse, 1996, Gutiérrez-Escribano and Nurse, 2015). To overcome this, we have also compared the relative contributions of activity thresholds and cyclin-substrate specificity in wild-type cells, where multiple cyclin-CDK complexes are expressed. Taken together, our findings demonstrate how activity thresholds order substrate phosphorylation and the downstream cell-cycle events, both in cells with a simplified CDK network and in wild-type cells with a multi-cyclin network.

## Results

### In Vivo CDK Substrates

**Figure 1:**
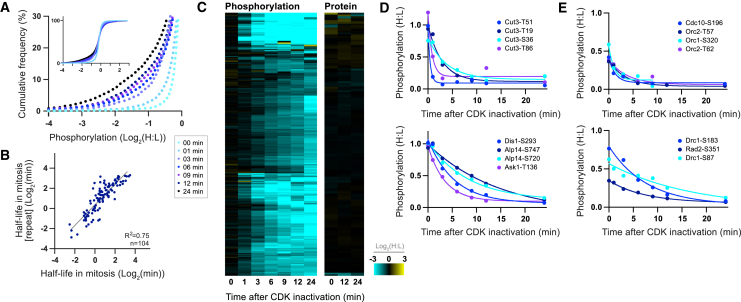
CDK Substrate Dephosphorylation after CDK Inactivation (A) The cumulative frequency of the relative phosphorylation of all detected phosphosites at time points after CDK inactivation in mitosis. (B) Plot of CDK substrate sites half-lives after CDK inactivation in mitosis against the half-lives calculated from a biological repeat (three sites with values < 15 s were excluded from this analysis). (C) Heatmap of CDK substrate site phosphorylation (n = 274) and protein (n = 146) levels after CDK inactivation in mitosis. Each row corresponds to a single phosphosite or protein, respectively. Rows are ordered by hierarchical clustering after imputation to replace missing values. Values outside the display range are set to the closest extreme. (D and E) The relative phosphorylation of individual CDK substrate sites after CDK inactivation in (D) mitosis and (E) S phase. Curves are a one-phase exponential decay fit to the data. See also Figure S1. See Figure S1A for experimental design. See STAR Methods for details of phosphorylation half-life calculation.

**Figure S1:**
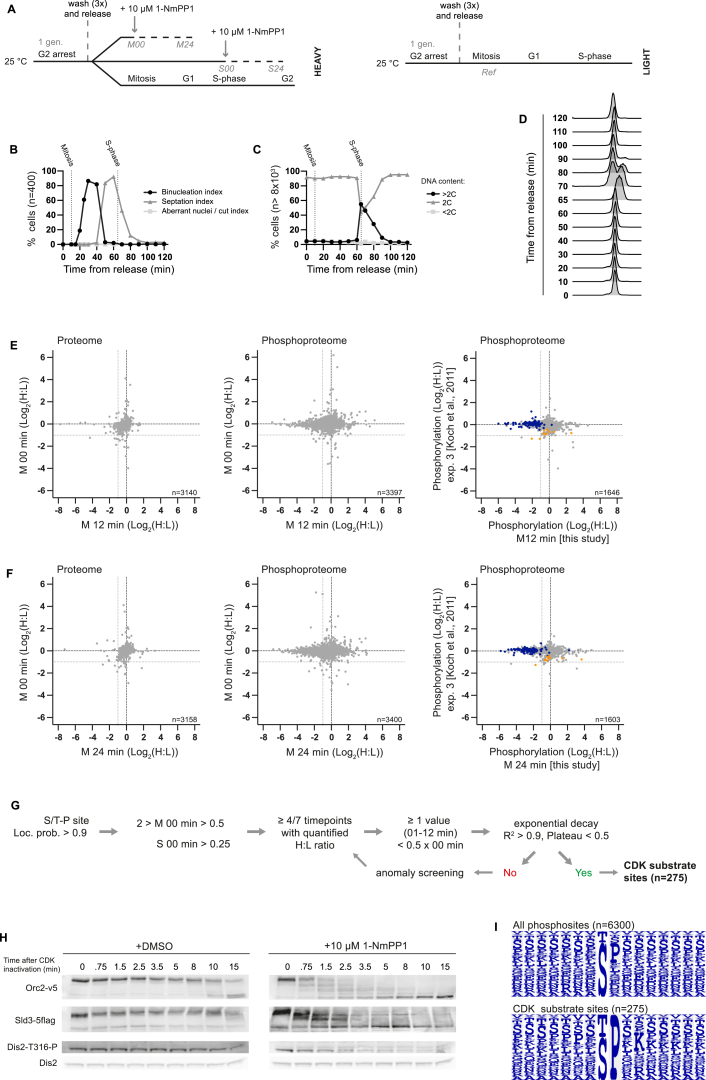
CDK Inactivation in Mitosis and S Phase and Defining CDK Substrates, Related to Figure 1 The phosphoproteome was analyzed after CDK inactivation in mitosis and S phase to define CDK substrates. (A) Schematic of experimental design: a heavy labeled culture (MS230) was released from G2 arrest. The culture was split and either treated with 10 μM 1-NmPP1 in mitosis (10 min after release) or S phase (65 min after release). Protein samples were taken at 0, 1, 3, 6, 9, 12 and 24 min after addition of 10 μM 1-NmPP1. Protein samples were mixed with a common light labeled reference (MS230, synchronized in mitosis). For the biological repeat protein samples were taken between 0 and 12 min after addition of 1-NmPP1 (MS122). (B-D) Cell-cycle progression was monitored after release from G2 arrest in untreated cells. (B) Quantification of chromosome and cell division. (C&D) DNA content profiles and quantification. (E&F) Scatterplot of the relative site phosphorylation or protein levels after CDK inactivation in mitosis ((E) 12 min after 1-NmPP1 treatment and (F) 24 min after 1-NmPP1 treatment). By 12 or 24 min after CDK inactivation in mitosis a significant proportion of the phosphoproteome is at least twofold decreased (13.2% and 16.9% respectively), whereas only a small fraction of the proteome is similarly decreased (1.25% and 0.78% respectively). This compares to 0.94% of the phosphoproteome and 0.53% of the proteome behaving similarly before CDK inactivation in mitosis (0 min). Right hand panels show a comparison with the phosphoproteome of cells without a *cdc2(as)* allele after 1-NmPP1 treatment in mitosis, reported by Koch et al. (2011). CDK substrate sites (blue) and sites defined by Koch et al. (2011) as being dephosphorylated due to non-specific 1-NmPP1 dependent effects (orange) are shown. (G) Workflow used to define CDK substrate sites (see STAR Methods for details). (H) western blot analysis of CDK substrate (Orc2, Sld3 and Dis2) phosphorylation in mitosis. Phosphorylation is monitored by mobility shift except for Dis2 where T316-P is directly detected. Cells (MS212) were synchronized in mitosis (10 min after G2 release) and treated with either 10 μM 1-NmPP1 or DMSO: see Figure S1A for experimental design. Protein samples were taken between 0 and 15 min after 1-NmPP1 or DMSO addition. Candidate CDK substrates are dephosphorylated between 10 and 15 min after DMSO treatment (i.e., without CDK inactivation). (I) Amino acid distribution surrounding all detected phosphosites and CDK substrate sites.

We defined in vivo CDK substrates by analyzing the phosphoproteome after inactivating CDK. Cells expressing an ATP analog-sensitive CDK allele were synchronized in mitosis or S phase, and CDK was inactivated by the addition of the ATP analog 1-NmPP1 (Bishop et al., 2000, Coudreuse and Nurse, 2010) (Figures S1A–S1D). Phosphoproteomic analysis of time-course samples after CDK inactivation in mitosis reveals a continuous decrease in global phosphorylation: 17% of phosphosites decreased more than 2-fold by 24 min, which could be either directly or indirectly downstream of CDK (Figure 1A). No major changes in global protein levels were detected (Figures S1E and S1F).

To rigorously define a class of direct CDK substrate sites, we identified phosphorylation events at the minimal CDK consensus sequence (S/T-P), which decreased more than 2-fold after CDK inactivation and whose dephosphorylation was immediate and continuous, fitting an exponential decay (n = 275) (Table S1) (Figures 1C–1E and S1G). These sites are enriched for the full CDK consensus site (+3 K/R) (Songyang et al., 1994) (Figure S1I) and do not overlap with non-specific 1-NmPP1 effects (Koch et al., 2011) (Figures S1E and S1F). It is possible a fraction of these sites are phosphorylated by kinases regulated very rapidly downstream of CDK. Phosphosites at the consensus site of other kinases do change in this experiment (e.g., Plk1 and Aurora), although the vast majority of these were not dephosphorylated immediately and did not fit an exponential decay (data not shown). Many of the proteins we identified have been previously characterized as CDK substrates, including orthologs of proteins with mitotic functions, such as SMC4 (Cut3), Cdc25, Plk1 (Plo1), Survivin (Bir1), INCENP (Pic1), Ask1, and TOG (Dis1 and Alp14), and proteins involved in DNA replication, such as Sld2 (Drc1), Sld3, Fen1 (Rad2), Orc1, and Orc2. Furthermore, these sites are enriched for cell-cycle-related GO categories as well as being enriched on the orthologs of human and *S. cerevisiae* CDK targets (Table S2) (Holt et al., 2009, Hornbeck et al., 2015). This, and the fact that these data can be used to derive half-lives that are quantitatively reproducible between biological repeats (Figure 1B), corroborates our conclusion that this set of 275 sites predominantly constitutes direct in vivo CDK substrates.

Previous large-scale proteomics studies have either defined putative CDK substrates in vitro (Blethrow et al., 2008, Chi et al., 2008, Errico et al., 2010, Ubersax et al., 2003) or have identified phosphorylation events downstream of CDK in vivo, which could be either direct or indirect substrates (Holt et al., 2009).

### CDK Substrate Phosphorylation Dynamics during the Cell Cycle

**Figure 2:**
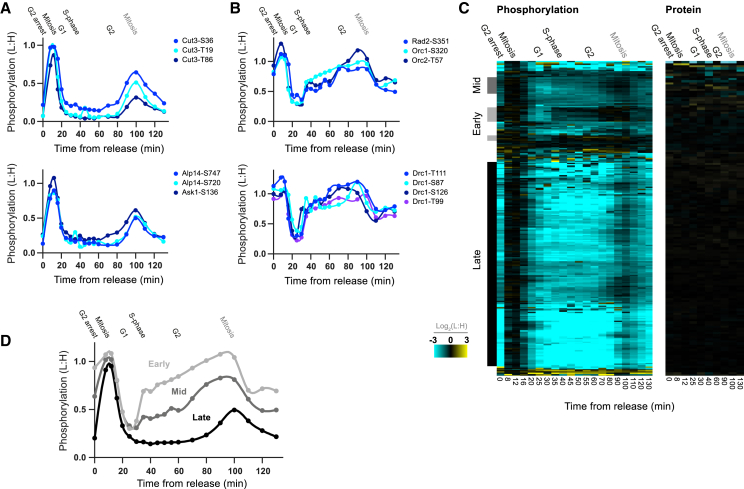
CDK Substrate Phosphorylation Dynamics during the Cell Cycle (A and B) The relative phosphorylation of individual CDK substrate sites after release from G2 arrest for mitotic (A) and S-phase (B) related proteins. Spline connects points. (C) Heatmap of CDK substrate site phosphorylation (n = 256) and protein (n = 149) levels after release from G2 arrest. Each row corresponds to a single phosphosite or protein, respectively. Rows are ordered according to hierarchical clustering, after imputation to replace missing values. Clusters of early (light gray, n = 16), mid (dark gray, n = 12), and late (black, n = 169) substrate sites are annotated. Values outside the display range are set to the closest extreme. (D) The median relative phosphorylation of early, mid, and late CDK substrate sites after release from G2 arrest. Spline connects points. See also Figure S2. See Figure S2A for experimental design.

**Figure S2:**
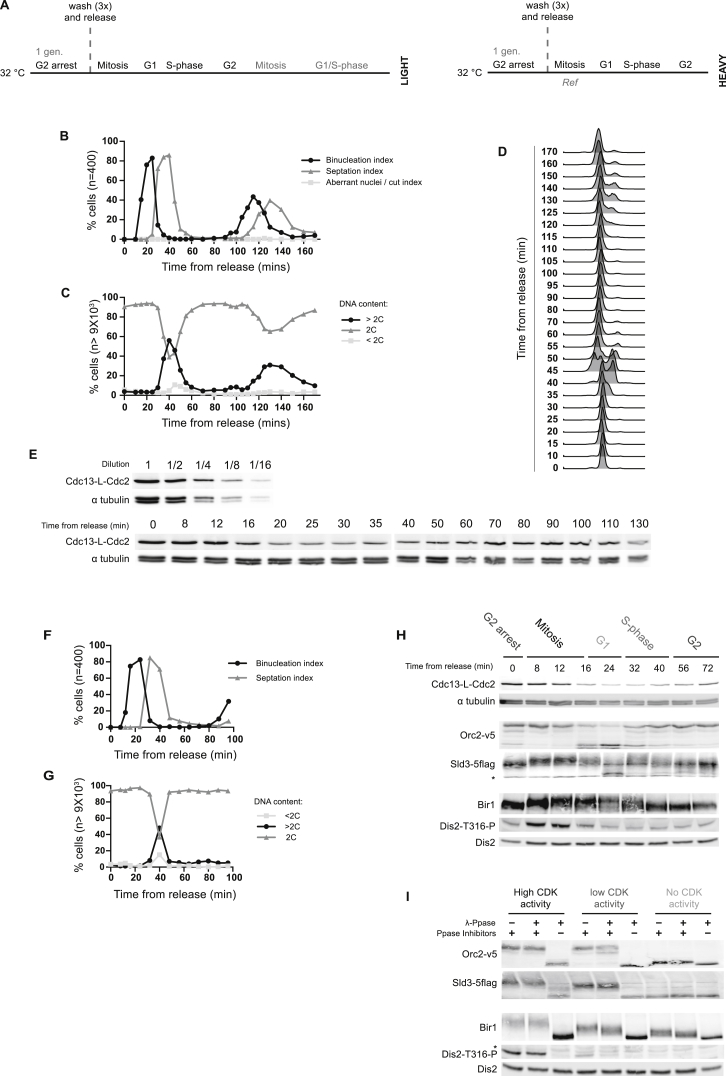
Cell-Cycle-Synchronized Cultures, Related to Figure 2 CDK substrate phosphorylation was analyzed during the cell cycle. (A) Schematic of experimental design: a light labeled culture (MS230) was released from G2 arrest and protein samples were recovered at 20 time points over the first and second cell division cycle. Protein samples were mixed with a common heavy labeled reference (MS230, synchronized in mitosis). (B-D) Cell-cycle progression was monitored after release from G2 arrest. (B) Quantification of chromosome and cell division. (C&D) DNA content profiles and quantification. (E) western blot analysis of Cdc13-L-Cdc2 protein levels after release from G2 arrest. (F-I) Candidate CDK substrate (Orc2, Sld3, Bir1 and Dis2) phosphorylation during the cell cycle was analyzed by western blotting. Phosphorylation was monitored by mobility shift except for Dis2, where T316-P was directly. Asterisks (^∗^) mark non-specific bands. Cells (MS132) were synchronized as in Figure S2A. (F&G) Cell-cycle synchrony after release from G2 arrest was monitored by quantification of (F) chromosome & cell division and (G) DNA content. (H) western blot analysis of Cdc13-L-Cdc2, α tubulin and the phosphorylation of candidate CDK substrates after release from G2 arrest. (I) Orc2, Sld3 & Bir1 mobility shifts and Dis2-T316-P signal are CDK and phosphorylation dependent. No CDK activity = 20 min 10 μM 1-NmPP1 treatment, low CDK activity = G2 arrest, and high CDK activity = synchronized in mitosis (MS132). Each sample was treated with: *(i)* mock, *(ii)* lambda phosphatase + phosphatase inhibitors or *(iii)* lambda phosphatase.

We then quantified the relative changes in the phosphorylation of these CDK substrates during two synchronized cell cycles following release from a G2 arrest (Figures 2 and S2A–S2E). Hierarchical clustering was used to identify three qualitatively distinct phosphorylation patterns during the cell cycle among CDK substrates: early, mid, and late (Figures 2C and 2D). Proteins phosphorylated early in the cell cycle show a stepwise increase in phosphorylation at the G1-to-S transition and continue to increase in phosphorylation across the cell cycle. By contrast, the majority of CDK substrate sites are first phosphorylated late in the cell cycle at G2/M. There is also a third category (mid substrates), which shows an increase in phosphorylation at both transitions, peaking at G2/M. All three categories are then simultaneously dephosphorylated at mitotic exit. CDK substrate protein levels do not change significantly over the cell cycle (Figure 2C). Early and late phosphorylation patterns were also observed by western blotting analysis of CDK substrate proteins (Figures S2F–S2H).

Early substrates are significantly enriched in GO terms relating to DNA replication, while late substrates are significantly enriched in GO terms relating to mitotic functions (Table S2). Early substrates only represent about one tenth of CDK substrate sites in *S. pombe*, which may reflect the relative simplicity in the processes of S phase as compared to mitosis, consistent with the ability to reconstitute eukaryotic DNA replication initiation in vitro (Yeeles et al., 2015). Interestingly, a number of early and late substrates identified here have no previously described CDK-regulated function and can now be putatively implicated in G1/S or mitotic functions, respectively. We also observed small differences in the phosphorylation dynamics of late substrates, suggesting that there is a more finely resolved temporal order among late substrate phosphorylation during the progression into and through mitosis (50–100 min after release) (Figure 2C).

These data show clearly resolved differences in the timing of substrate phosphorylation as the cell cycle progresses and that sites phosphorylated earlier continue to increase until the end of the cell cycle, when all substrates are dephosphorylated together. This sequential temporal ordering is striking, considering that all of these substrates are phosphorylated by the same cyclin-CDK (Cdc13-L-Cdc2). To understand how these patterns in phosphorylation are achieved, we set out to assay the changes in this CDK activity during the cell cycle.

### Substrate Phosphorylation Rates as a Measure of the In Vivo CDK-to-Phosphatase Activity Ratio

**Figure 3:**
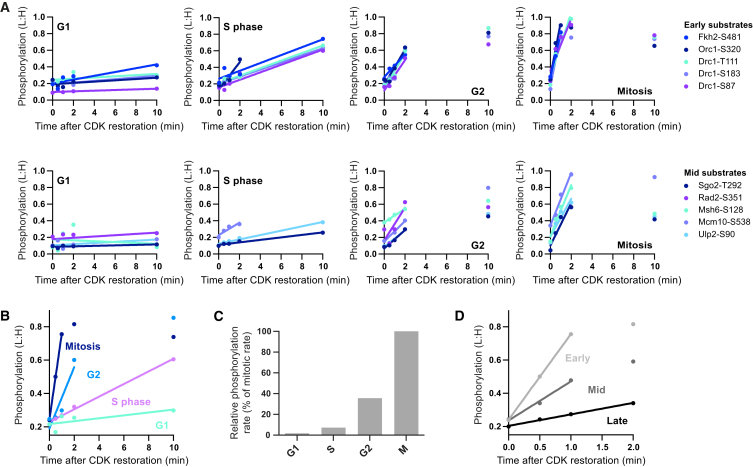
In Vivo Substrate Phosphorylation Rates as a Measure for the CDK-to-Phosphatase Activity Ratio (A) The relative phosphorylation of individual early and mid CDK substrate sites after transient CDK inactivation and then restoration in G1, S phase, G2, and mitosis. (B) The median relative phosphorylation of early CDK substrate sites after CDK restoration in G1, S phase, G2, and mitosis. (C) The relative phosphorylation rates (% of mitotic rates) calculated from the slope of the linear regressions in (B). (D) The median relative phosphorylation of early, mid, and late CDK substrate sites after CDK restoration in mitosis. See also Figures S3A–S3D. See Figure S3A for experimental design. See STAR Methods for details of phosphorylation rate calculation.

**Figure 4:**
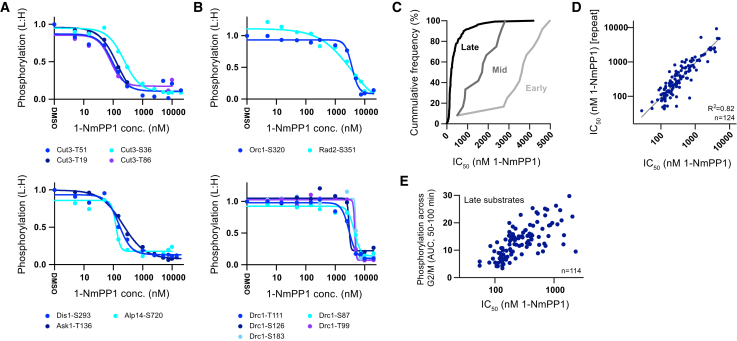
In Vivo Substrate Sensitivity to CDK Activity (A and B) The relative phosphorylation of individual CDK substrate sites over a range of 1-NmPP1 concentrations for mitotic (A) and S-phase (B) related proteins (normalized so L:H [DMSO] = 1). Curves are a four-parameter sigmoidal fit to the data. (C) The cumulative frequency of 1-NmPP1 IC_50_ values for early (n = 12), mid (n = 12), and late (n = 116) CDK substrate sites. (D) Plot of CDK substrate site 1-NmPP1 IC_50_ values from biological repeats. Two values below the axes limit are excluded from this analysis. (E) Plot of 1-NmPP1 IC_50_ values against total phosphorylation during G2/M (AUC, 50–100 min) for late CDK substrate sites. Two values are below the axis limits. See also Figures S3E–S3J. See Figure S3E for experimental design. See STAR Methods for details of IC_50_ and AUC calculation.

**Figure S3:**
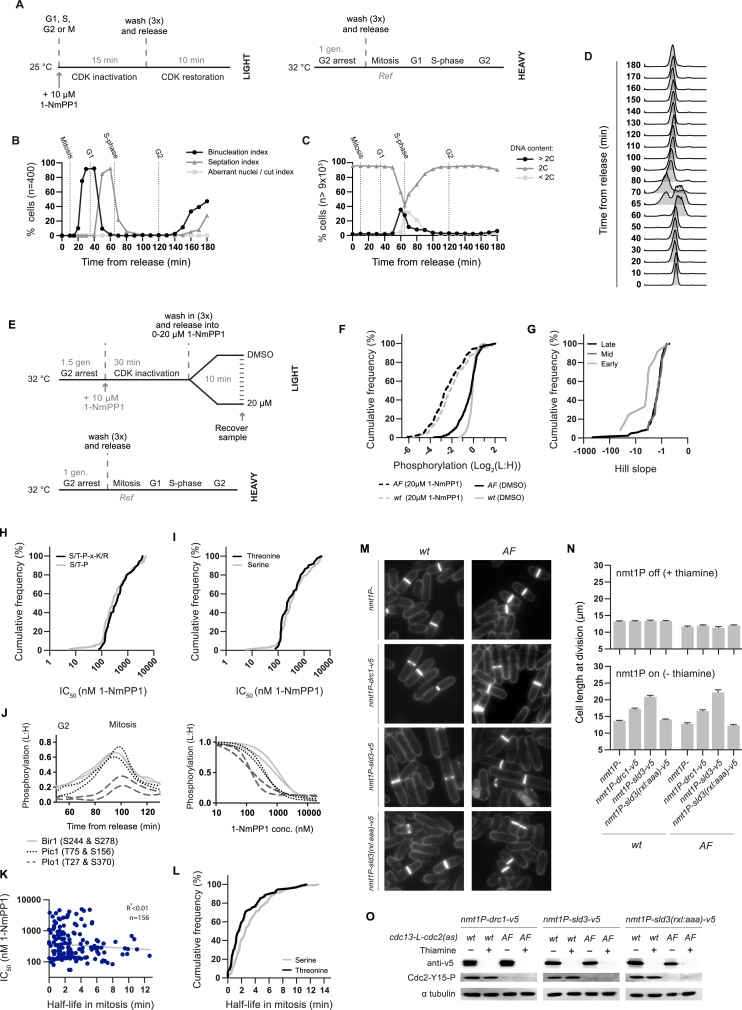
CDK Substrate Phosphorylation Rates, Dephosphorylation Rates, and Sensitivity to CDK Activity, Related to Figures 3, 4, and 5A–5F (A–D) Related to Figure 3. CDK was transiently inactivated at different cell-cycle stages and CDK substrate re-phosphorylation rates were quantified as a proxy for the CDK-to-phosphatase activity ratio. (A) Schematic of experimental design: a light labeled culture (MS213) was synchronized as in Figure S1A. In mitosis (10 min after release), G1 (35 min after release), S phase (65 min after release) or late G2 (120 min after release) a parallel culture was treated with 10μM 1-NmPP1 to inactivate CDK (15 min). 1-NmPP1 was then washed out (3x wash, 40 s per wash) to restore CDK activity. Protein samples were taken before (0 min) and 0.5, 1, 2 and 10 min after completion of the final wash. Protein samples were mixed with a common heavy labeled reference (MS230, synchronized in mitosis). (B-D) Cell-cycle progression was monitored after release from G2 arrest in untreated cells. (B) Quantification of chromosome and cell division. (C&D) DNA content profiles and quantification. (E–J) Related to Figure 4. Cells were arrested and released into a range of 1-NmPP1 concentrations to quantify CDK substrate phosphorylation across a range of CDK activity levels. (E) Schematic of experimental design: a light labeled cultures were arrested for 1.5 generations (1 generation in 1μM 1-NmPP1 followed by 0.5 generations in 2μM 1-NmPP1) and CDK was then inactivated by the addition of 10 μM 1-NmPP1. Cultures were then washed in and released into media containing DMSO (0.2%) or 1-NmPP1 (5 nM, 15 nM, 50 nM, 150 nM, 300 nM, 1 μM, 2.5 μM, 5 μM, 7.5 μM, 10 μM or 20 μM). Protein samples were taken 10 min after release and were mixed with a common heavy labeled reference (MS230, synchronized in mitosis). An *AF* strain (T14A, Y15F mutations in the Cdc2 moiety) (MS86) was used to bypass feedback on CDK activity. Protein samples were also recovered from a strain with a wild-type (*wt*) Cdc2 moiety (MS87) in DMSO and 20 μM 1-NmPP1. *rad3Δ* was introduced into the *wt* control to ensure that DNA replication/damage checkpoint signaling did not cause T14/Y15 phosphorylation dependent CDK inhibition. (F) The cumulative frequency (% of CDK substrate sites) of the relative phosphorylation values in *AF* and *wt* after release into DMSO or 20 μM 1-NmPP1. There is a major global increase in CDK substrate site phosphorylation between the extremes of the titration series for both *AF* and *wt*. *AF* (DMSO) n = 236, *AF* (20 μM 1-NmPP1) n = 218, *wt* (DMSO) n = 242, *wt* (20 μM 1-NmPP1) n = 220. (G) The cumulative frequency of the 1-NmPP1 Hill slope values for early, mid and late CDK substrate sites. Median Hill slope values are −3.579, −1.325 and, −1.406 for early (n = 12), mid (n = 12) and late (n = 117) substrate sites respectively. See STAR Methods for details of Hill slope value calculation. (H and I) The cumulative frequency of 1-NmPP1 IC_50_ values for CDK substrate sites at (H) the minimal or full CDK consensus sequence and (I) serine or threonine phosphosites. See STAR Methods for details of IC_50_ value calculation. (J) The relative phosphorylation of individual CDK substrate sites in Bir1, Pic1 and Plo1 during G2/M (50-100 min after release from G2 arrest, only Spline plotted for presentation) and over a range of 1-NmPP1 concentrations (only sigmoidal fit plotted for presentation). (K and L) Related to Figures 5A and 5B. (K) Plot of phosphorylation half-lives after CDK inactivation in mitosis against 1-NmPP1 IC_50_ values for CDK substrate sites. Three values below the axis limits are not shown. See STAR Methods for details of IC_50_ and phosphorylation half-life calculation. (L) The cumulative frequency of phosphorylation half-lives after CDK inactivation in mitosis for serine and threonine CDK substrate sites. See STAR Methods for details of phosphorylation half-life calculation. (M–O) Related to Figure 5F: Drc1-v5, Sld3-v5 and Sld3(RxL:AAA)-v5 were expressed from the full strength nmt1 promoter in cells expressing a wild-type (*wt*) or checkpoint insensitive (*AF*) Cdc2 moiety (MS312-319). Cells were initially cultured in EMM4S + thiamine. Thiamine washout was used to induce expression from the nmt1 promoter. (M) Representative photos of calcofluor stained cells 48 hr after thiamine washout. (N) Cell length measurements (mean + S.E.M., n = 50) of dividing cells before (top panel) and 48 hr after (bottom panel) thiamine washout. NB bottom panel is reproduced in Figure 5F. (O) western blot analysis of Drc1-v5, Sld3-v5 and Sld3(RxL:AAA)-v5 expression before and 48 hr after thiamine washout.

Various estimates of CDK activity during the cell cycle have been reported across different eukaryotic systems. These have mostly measured the rate of phosphorylation of a model substrate in vitro (Draetta and Beach, 1988, Labbé et al., 1988, Moreno et al., 1989). More relevant, however, is the in vivo balance in activity between CDK and counteracting phosphatase(s). This has been previously estimated by measuring the phosphorylation level of model synthetic fluorescent reporters (Gavet and Pines, 2010a, Gavet and Pines, 2010b, Spencer et al., 2013) but, given that any particular substrate can exist in a net phosphorylated or unphosphorylated state over a wide range of CDK activity levels (Figure 4), measuring the steady state phosphorylation level of a single model substrate may not accurately reflect all changes in kinase activity. In contrast, measuring the phosphorylation rate of multiple substrates (e.g., for early or mid substrates that are phosphorylated throughout most of the cell cycle) should provide a more direct and comprehensive measure. We have developed a new approach, using phosphoproteomics to quantify substrate phosphorylation rates in vivo, to estimate the relative changes in the CDK-to-phosphatase activity ratio. Cells were synchronized at different cell-cycle stages (G1, S phase, G2, and mitosis) and CDK was transiently inactivated to allow substrate dephosphorylation. 1-NmPP1 was then washed out to restore CDK activity (Figures S3A–S3D). Instantaneous CDK substrate phosphorylation rates were obtained after the restoration of CDK activity by analyzing the period in which the phosphorylation of any given substrate site increased approximately linearly (Figure 3A).

Figures 3B and 3C show that the relative rate of early substrate phosphorylation increases as cells move from G1-to-S, from S-to-G2, and to G2-to-M, indicating that CDK activity rises continuously throughout the cell cycle. The low interphase activity levels rise incrementally from 1.7% of mitotic levels in G1, to 7.3% in S phase, and then 36% in G2 before peaking in mitosis (100%). These measurements show that CDK activity is more than 10-fold greater in mitosis than S phase, indicating that the patterns of substrate phosphorylation presented in Figure 2 could be achieved by a differential sensitivity among substrates to this rising CDK activity.

### Substrate Sensitivity to In Vivo CDK Activity

To test this directly, we quantified the relative sensitivity of CDK substrate sites to CDK activity by releasing arrested cells into a range of CDK inhibitor (1-NmPP1) concentrations and analyzing the phosphoproteome (Figures 4A–4D and S3E). This allowed us to quantify the extent of substrate phosphorylation at different in vivo CDK activity levels, independently of differences in the cell-cycle history of the cells. CDK activation is non-linear due to positive and double-negative feedback loops that act via the inhibitory T14/Y15 phosphorylation on CDK (Pomerening et al., 2005, Solomon et al., 1990). To ensure that the output CDK activity was a more linear function of 1-NmPP1 concentration, a genetic background that bypasses these auto-regulatory feedback loops was used (*cdc13-L-cdc2AF(T14AY15F)*) (Coudreuse and Nurse, 2010). Figures 4A–4D show that early substrates are phosphorylated at significantly higher 1-NmPP1 concentrations, and thus lower CDK activity levels, than late substrates. Mid substrates show an intermediate response. The median half maximal inhibition concentration (IC_50_) of 1-NmPP1 values are 3.7 μM, 1.7 μM, and 0.2 μM for early, mid, and late substrates, respectively. Consistent with this, later substrates are re-phosphorylated slower than earlier substrates after transient CDK inactivation in mitosis (Figure 3D). These data demonstrate that the timing of a substrate’s phosphorylation during the cell cycle correlates with its sensitivity to CDK activity: substrates phosphorylated early in the cycle become phosphorylated at lower CDK activity levels than late substrates.

There is also a correlation among late substrates between their phosphorylation during G2/M and their sensitivity to CDK activity (Figures 4E and S3J), meaning that small differences in the timing of late substrate phosphorylation may occur in a CDK activity-dependent manner. We note that the increase in phosphorylation of early substrates is more abrupt (i.e., have more negative Hill slopes) and that the global differences in substrate sensitivities do not appear to be related to a preference for the full CDK consensus site or a major bias toward serine or threonine (Figures S3G–S3I).

### Substrate Dephosphorylation Rates and Putative Cyclin Docking Motifs

**Figure 5:**
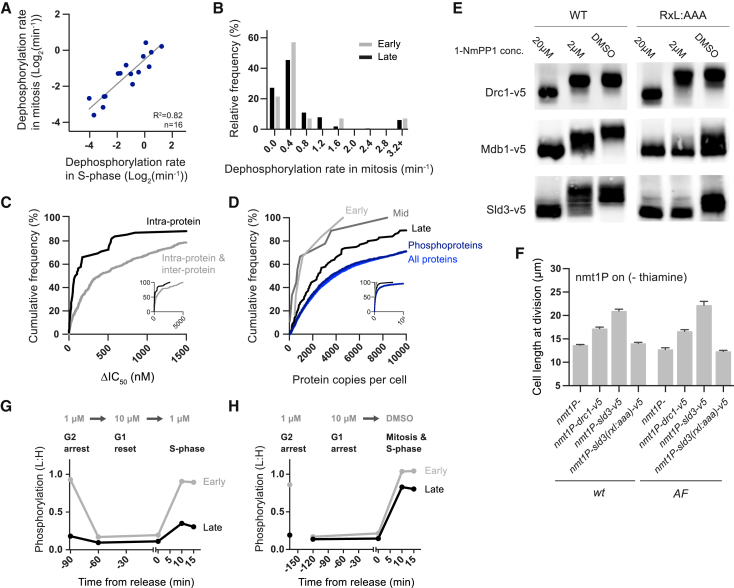
CDK Substrate Dephosphorylation Rates, Cyclin-Docking Motifs, and Substrate Phosphorylation during Reordered Cell-Cycle Experiments (A and B) Dephosphorylation rates. (A) Plot of the CDK substrate site dephosphorylation rates after CDK inactivation in S phase against in mitosis. Three phosphosites with values < 15 s were excluded. (B) Histogram of the relative frequency of dephosphorylation rates after CDK inactivation in mitosis for early and late CDK substrate sites. See also Figures S3K and S3L. See Figure S1A for experimental design. See STAR Methods for details of dephosphorylation rate calculation. (C) The cumulative frequency of the difference between the 1-NmPP1 IC_50_ values (ΔIC_50_) for all phosphosite pairs (inter- and intra-protein comparisons, n = 1711) and phosphosite pairs in the same protein (intra-protein comparisons, n = 62). ΔIC_50_ values were only calculated for pairs of phosphosites from substrates with more than one phosphosite IC_50_ value. ΔIC_50_ populations are significantly different (p < 0.001, two tailed Mann-Whitney U test). (D) The cumulative frequency of the protein copies per cell (Marguerat et al., 2012) for early (n = 6), mid (n = 9), and late (n = 100) CDK substrates; all phosphoproteins detected in this study (n = 1209); and all proteins (n = 3301). 178 values are above the axis limit. (E) Western blot analysis of candidate early substrate (Sld3, Drc1, and Mdb1) phosphorylation. Phosphorylation is monitored by mobility shift. Wild-type (WT) and putative cyclin docking motif mutant (RxL:AAA) versions of Drc1, Mdb1, and Sld3 were analyzed after release into DMSO, 2 μM or 20 μM 1-NmPP1 (MS306-311). See Figure S3E for experimental design. (F) Cell length at division (mean + SEM, n = 50) after Drc1, Sld3, or Sld3(RxL:AAA) overexpression in *wt* or checkpoint-insensitive *AF* strains. See also Figures S3M–S3O. (G and H) Median relative phosphorylation of early and late CDK substrate sites during two reordered cell cycles. Cell cycle stages and 1-NmPP1 concentrations are denoted above the graphs. See also Figures S4, S5, and S6. See Figure S4A for experimental design.

It has been speculated that a more rapid phosphatase-dependent turnover of specifically late substrate phosphorylation prevents their net phosphorylation at lower CDK activity levels (Coudreuse and Nurse, 2010, Fisher et al., 2012, Uhlmann et al., 2011). We tested this possibility by calculating the dephosphorylation rates of early and late substrate sites after CDK inactivation in mitosis and S phase. For substrates phosphorylated at both stages (e.g., early substrates), there are no substantial changes in the dephosphorylation rates between S phase and mitosis, suggesting that these sites are not subject to a phosphatase activity that is differentially regulated between these two conditions (Figure 5A). Second, there is no significant difference in the dephosphorylation rate between early and late substrates and no correlation between phosphorylation half-life and 1-NmPP1 IC_50_ (Figures 5B and S3K). These data indicate that early and late substrates are not subject to differing dephosphorylation regimes in these experimental conditions. We note that CDK-phosphorylated threonines are dephosphorylated somewhat faster than serines (Figure S3L).

To explore how the sensitivity of a substrate site is established, we analyzed proteins in which we identified multiple CDK substrate sites. Figure 5C shows that the pairwise differences in 1-NmPP1 IC_50_ values (ΔIC_50_) between sites on the same protein are significantly smaller than those between sites on different proteins, demonstrating that sites on the same protein behave more similarly to one another. We also note that early substrates tend to be less abundant than late CDK substrates (Marguerat et al., 2012) (Figure 5D). This could reflect the possibility that CDK substrate sensitivity is not set independently for each site, but is determined at the level of the whole protein by a substrate’s affinity for cyclin-CDK. To investigate this possibility, we mutated the putative cyclin-docking motifs (RxL motifs) in three candidate early substrates: Drc1, Sld3, and Mdb1. Figure 5E shows that, while the wild-type versions of Sld3 and Mdb1 are hyper-phosphorylated at the low (2 μM 1-NmPP1) and high (DMSO) CDK activity levels, Sld3(RxL:AAA) and Mdb1(RxL:AAA) only become phosphorylated in DMSO. There is no such difference between Drc1 and Drc1(RxL:AAA) (Figure 5E). Therefore, the disruption of the RxL motif in Sld3 and Mdb1 effectively transforms them from early into late substrates, suggesting that the increased sensitivity of early substrates to CDK activity may be achieved, at least in part, via an enhanced affinity for cyclin-CDK. We also observed that the strong overexpression of Drc1 or Sld3, but not Sld3(RxL:AAA), causes a checkpoint-independent cell-cycle delay, resulting in increased cell length at division (Figures 5F and S3M–S3O).

Taken together, our data show that the timing of a substrate’s phosphorylation does not correlate with its in vivo dephosphorylation rate, but it does correlate with its sensitivity to in vivo CDK activity. In conjunction with our measurements of CDK activity at different cell cycle stages, this supports a model where a single rising CDK activity orders substrate phosphorylation via the sequential passage through substrate-specific activity thresholds and that this, in turn, orders cell cycle events.

### Reordering Substrate Phosphorylation and the Cell Cycle

**Figure S4:**
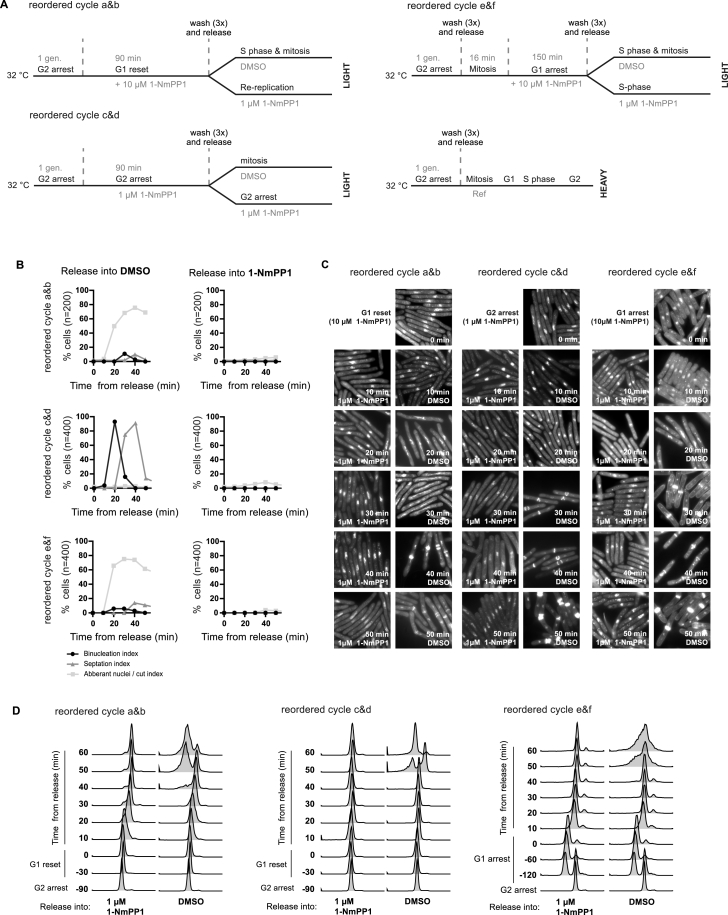
Reordering S Phase and Mitosis, Related to Figures 5G and 5H (A–D) S phase and mitosis were reordered using 1-NmPP1 treatment regimes as previously described (Coudreuse and Nurse, 2010). (A) Schematic of experimental design: light labeled cultures were reset in G1 (reordered cycle a&b (MS230)), arrested in G2 (reordered cycle c&d (MS230)) or arrested in G1 (reordered cycle e&f (MS108)). Cultures were then washed and released into DMSO (reordered cycle a, c and e) or 1 μM 1-NmPP1 (reordered cycle b, d and f). Protein samples were taken during the arrest or reset and 10 & 15 min after release. Protein samples were mixed with a common heavy labeled reference (MS230, synchronized in mitosis). (B-D) Cell-cycle progression was monitored during reordered cycle a-f. (B) Quantification of chromosome and cell division. (C) Representative photos of DAPI (DNA) and calcofluor (cell septum) stained cells. Aberrant nuclei and cut cells reflect mitotic progression concurrent with ongoing DNA synthesis (Coudreuse and Nurse, 2010). (D) DNA content profiles.

**Figure S5:**
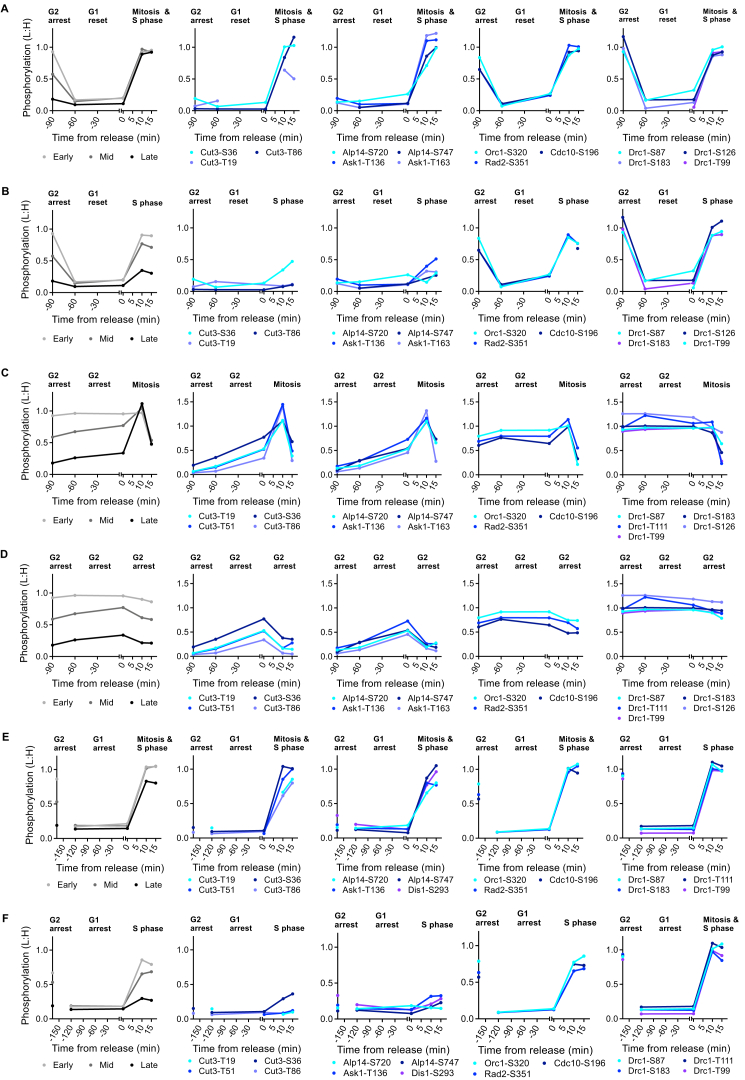
CDK Substrate Phosphorylation during Six Reordered Cell-Cycle Experiments, Related to Figures 5G and 5H (A–F) CDK substrate phosphorylation during reordered cell cycle a-f, respectively. Reordered cycles a-f are outlined in Figure S4 and cell-cycle stages are annotated above the graphs. Left hand panel shows the median relative phosphorylation of early mid and late CDK substrate sites. Remaining four panels shows the relative phosphorylation of individual CDK substrate sites with examples of protein with S-phase-related and mitotic functions presented in separate panels. See Figure S4 for experimental design. NB data in Figures S5B and S5E are reproduced in Figures 5G and 5H.

It has previously been reported that altering CDK activity can reorder S phase and mitosis, indicating there is no intrinsic order to these events during the *S. pombe* cell cycle (Coudreuse and Nurse, 2010). We have analyzed the phosphoproteome during six different reordering experiments (Figures S4 and S5). If the ordering of CDK substrate phosphorylation is indeed critical for the ordering of downstream cell-cycle events, then the relationship between CDK activity, substrate phosphorylation, and cell-cycle stage should be maintained when the canonical sequence of cell-cycle events is altered during these experiments.

**Figure S6:**
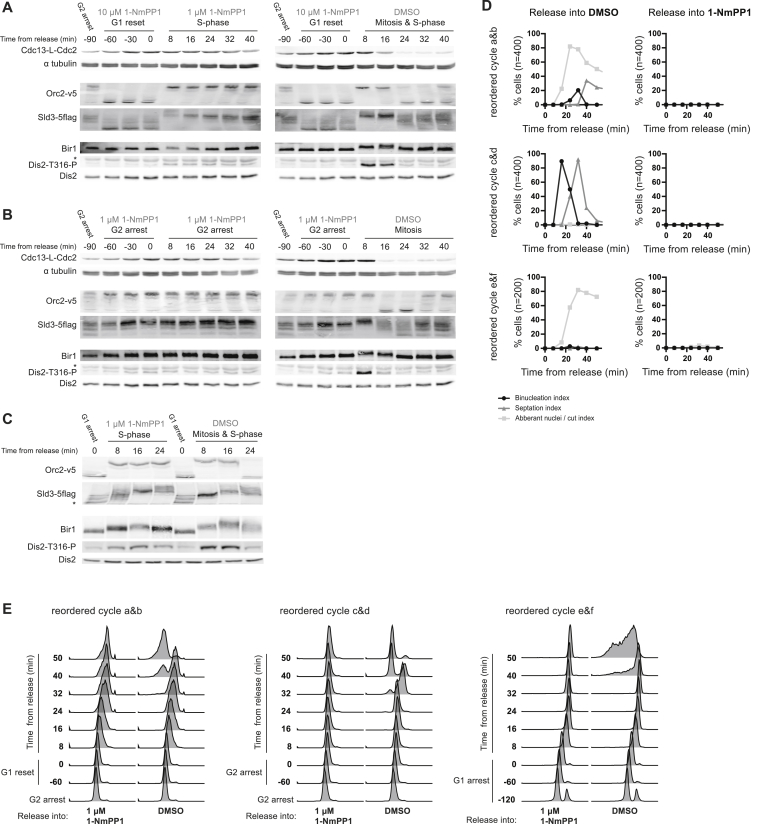
Candidate CDK Substrate Phosphorylation during Six Reordered Cell-Cycle Experiments, Related to Figures 5G and 5H (A–C) western blot of Cdc13-L-Cdc2, α tubulin and the phosphorylation state of four CDK substrates (Orc2, Sld3, Bir1 and Dis2) during reordered cycle a-f respectively. See Figure S4a for experimental design. Phosphorylation is monitored by mobility shift, except for Dis2, where T316-P is directly detected. Asterisk (^∗^) mark non-specific bands. (D&E) Cell-cycle progression was monitored during reordered cycle a-f. (D) Quantification of chromosome and cell division. (E) DNA content. NB for reordered cycle a-d, cells were cultured in YE4S (MS212) due to *orc2-v5* dependent retardation of re-replication in EMM4S. For reordered cycles e&f, cells were cultured in EMM4S (MS132).

The relative phosphorylation of early and late substrate sites during two of these six reordering experiments is shown in Figures 5G and 5H. First, when CDK is inactivated (10 μM 1-NmPP1) in G2, early substrates become dephosphorylated. If an intermediate CDK activity level is subsequently restored (1 μM 1-NmPP1), early substrates, but not late substrates, are re-phosphorylated and a single round of DNA synthesis takes place (Figures 5G and S5B). This illustrates that the dephosphorylation and re-phosphorylation of early substrates, in and of itself, is sufficient to initiate a single round of DNA synthesis from G2. Second, cells arrested in G1 (10 μM 1-NmPP1) accumulate inactive Cdc13-L-Cdc2 so early and late substrates remain unphosphorylated. If these cells are then exposed to high levels of CDK activity (DMSO), both early and late substrates become phosphorylated, and G1 cells enter S phase and mitosis simultaneously (Figures 5H and S5E). Thus, simply via the premature phosphorylation of late substrates, G1 cells can be precociously forced into mitosis. CDK substrate site phosphorylation during all six reordering experiments is shown in Figure S5. These patterns were also observed by western blotting analysis of CDK substrate proteins (Figure S6).

These data demonstrate that, when the order of CDK substrate phosphorylation is perturbed, there is a corresponding reordering of the cell cycle, supporting a causal relationship between CDK activity thresholds, differential substrate phosphorylation, and the initiation of the G1-to-S and G2-to-M transitions.

### Comparative Analysis with Wild-Type Cells Expressing Multiple Cyclin-CDK Complexes

All of the experiments above were conducted in the simplified CDK network. In wild-type *S. pombe*, CDK differentially associates with Cdc13 and three other non-essential cyclins that function during G1 and S phase: Cig1, Cig2, and Puc1 (Bueno et al., 1991, Connolly and Beach, 1994, Fisher and Nurse, 1996, Martín-Castellanos et al., 2000, Mondesert et al., 1996, Obara-Ishihara and Okayama, 1994). It could be argued that CDK activity thresholds are only responsible for ordering substrate phosphorylation in cells run on the simplified system, where G1/S cyclin-CDK complexes are absent. To evaluate the contribution of G1/S cyclin-CDK complexes, we quantified CDK substrate phosphorylation in wild-type cells and compared this with the phosphorylation dynamics in cells deleted for all three G1/S cyclins (*ΔCCP*: *cig1Δ cig2Δ puc1Δ*). Because *ΔCCP* cells typically have an extended G1 that is dependent on the CDK inhibitor Rum1 (Martín-Castellanos et al., 2000), we also analyzed CDK substrate phosphorylation in *cig1Δ cig2Δ puc1Δ rum1Δ* cells.

**Figure 6:**
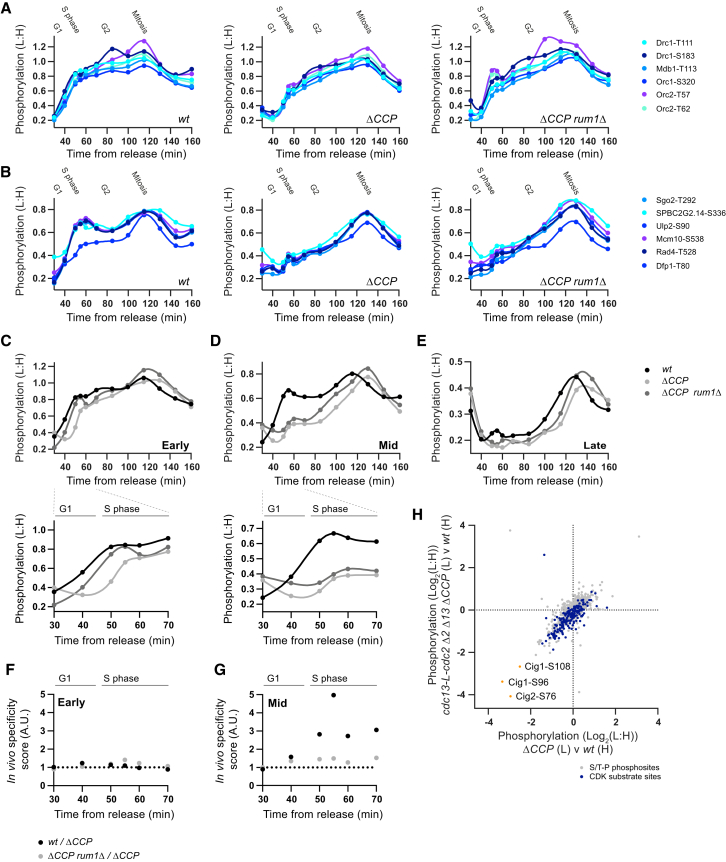
Comparative Analysis with Wild-Type Cells Expressing Multiple Cyclin-CDK Complexes (A and B) The relative phosphorylation of individual (A) early and (B) mid CDK substrate sites, after release from G2 arrest, in wild-type cells (*wt*) and cells deleted for G1/S cyclins (*ΔCCP* and *ΔCCP rum1Δ*). Spline connects points. Imputation to replace missing values and data smoothing were applied. NB sites in Rad4 and Dfp1 were redefined as mid substrates after visual inspection of the data. (C–E) The median relative phosphorylation of early, mid, and late CDK substrate sites after release from G2 arrest in *wt* (C), *ΔCCP* (D), and *ΔCCP rum1Δ* (E) cells. (F and G) Plot of the G1/S cyclin specificity score for (F) early and (G) mid substrates. (H) The relative phosphorylation of all detected S/T-P phosphosites (n = 1279) (gray), including CDK substrate sites (n = 170) (dark blue), and G1/S cyclin phosphosites (orange) in *ΔCCP* and *cdc13-L-cdc2 Δ2 Δ13 ΔCCP* cells during S-phase arrest. Sites for which 0.5 > L:H > 2 in a *wt* versus *wt* control were excluded. See also Figure S7. See Figures S7A and S7E for experimental design. See STAR Methods for G1/S cyclin-specificity score calculation.

**Figure S7:**
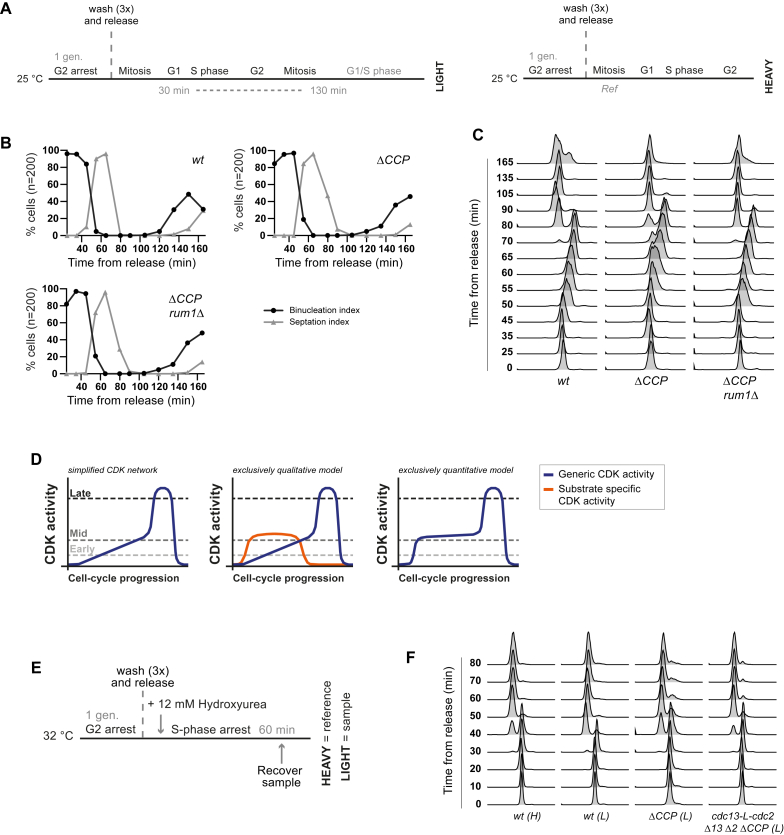
Comparative Analysis with Wild-Type Cells Expressing Multiple Cyclin-CDK Complexes, Related to Figure 6 (A–D) Related to Figures 6A–6G: CDK substrate phosphorylation was analyzed during the cell cycle in the presence and absence of G1/S cyclins. (A) Schematic of experimental design: light-labeled wild-type (*wt*) (MS282), *cig1Δ cig2Δ puc1Δ* (*ΔCCP*) (MS278) and *cig1Δ cig2Δ puc1Δ rum1Δ* (*ΔCCP rum1Δ*) (MS69) cultures were release from G2 arrest. To avoid complications regarding SILAC media differentially influencing the cell-cycle distribution among the above strains, light labeled samples for all three strains were grown in EMM4S. Protein samples were recovered between 30 min (first G1) and 160 min (second G1) and were mixed with a common heavy labeled reference (MS131, synchronized in mitosis). (B&C) Cell-cycle progression was monitored after release from G2 arrest. (B) Quantification of chromosome and cell division. (C) DNA content profiles. (D) Schematics illustrating how CDK activity orders substrate phosphorylation in the minimal CDK network (left panel) and two hypothetical models for a multi-cyclin system (center and right panel). An exclusively qualitative model involves phosphorylation at G1/S occurring due to G1/S cyclin-substrate specify and high generic CDK activity initiating mitosis (middle panel). In contrast, an exclusively quantitative model involves G1/S cyclins only modifying the profile in the rise of total generic CDK activity to promote phosphorylation at G1/S. (E–F) Related to Figure 6H. The phosphoproteome was analyzed during S-phase arrest in the presence and absence of G1/S cyclins. (E) Schematic of experimental design: light labeled *wt* (MS131), *ΔCCP* (MS200) and *cdc13-L-cdc2 Δ13 Δ2 ΔCCP* (MS213) cell were released from G2 arrest and treated with 12mM Hydroxyurea (HU) 10 min after release. Protein samples were recovered during the subsequent S-phase arrest (60 min after release) and mixed with a heavy labeled *wt* (MS131) protein sample also taken during S-phase arrest. (F) DNA content profiles during S-phase arrest.

Figures 6A–6E shows the phosphorylation of CDK substrates during the cell cycle in these different genetic backgrounds. The presence of G1/S cyclins (*wt*) somewhat sharpens the rise in early substrate phosphorylation across G1/S (Figures 6A and 6C), but more obviously causes an advancement in the timing of the phosphorylation of mid substrates (Figures 6B and 6D). Mid substrate phosphorylation rises gradually across the entire cell cycle in *ΔCCP* cells, but increases sharply at the G1-to-S transition in the presence of G1/S cyclins. The differences between wild-type and *ΔCCP* could be achieved either by G1/S cyclin-CDKs contributing additional generic CDK activity to modify the profile in the rise of total activity or by having an enhanced specificity toward early and/or mid substrates (Figure S7D). We propose that G1/S cyclins regulate early substrates by the former mechanism and mid substrates by the latter. This is because, in the presence of G1/S cyclins there is only a modest increase in early substrate phosphorylation, accompanied by a modest increase in late substrate phosphorylation in interphase, consistent with G1/S cyclin-CDKs providing generic activity (Figures 6C and 6E). Furthermore, the minor delay in early substrate phosphorylation in *ΔCCP* is mostly rescued by the deletion of the CDK inhibitor *rum1* gene (Figures 6A and 6C). In contrast, mid substrate phosphorylation is significantly delayed in the absence of G1/S cyclins, even when *rum1* is deleted (Figures 6B and 6D). To further test this interpretation, we calculated a G1/S cyclin specificity score by interpolating the phosphorylation values in Figures 6C–6E from the 1-NmPP1 dose-response experiment (Figure 4) (see STAR Methods for details). During S phase, mid substrates have a specificity score greater than one, meaning that their phosphorylation corresponds to a lower 1-NmPP1 concentration (i.e., higher CDK activity) than other CDK substrates when G1/S cyclins are present (Figure 6G). This is not the case for early substrates (Figure 6F), consistent with the conclusion that early substrates are not subject to substantial G1/S cyclin specificity, but that mid substrates are.

It is conceivable that some CDK substrates may not have been identified in our analysis because only G1/S cyclin-CDKs can phosphorylate them. To investigate this, we analyzed the phosphoproteome of wild-type and *ΔCCP* cells during an S-phase arrest. Phosphosites in Cig1 and Cig2 are significantly decreased in *ΔCCP* cells, but we found no S/T-P (minimal CDK consensus) phosphosites that decreased to a similar extent. This means that if G1/S cyclin-unique substrates do exist in *S. pombe*, then they can only represent a very small fraction of CDK substrate sites (Figure 6H).

## Discussion

Here, we report a phosphoproteomics-based systems analysis of CDK substrate phosphorylation and demonstrate the importance of substrate-specific CDK activity thresholds for the temporal ordering of S phase and mitosis, using the model eukaryote *S. pombe* (fission yeast).

### The Temporal Ordering of Substrate Phosphorylation in a Simplified CDK Network

**Figure 7:**
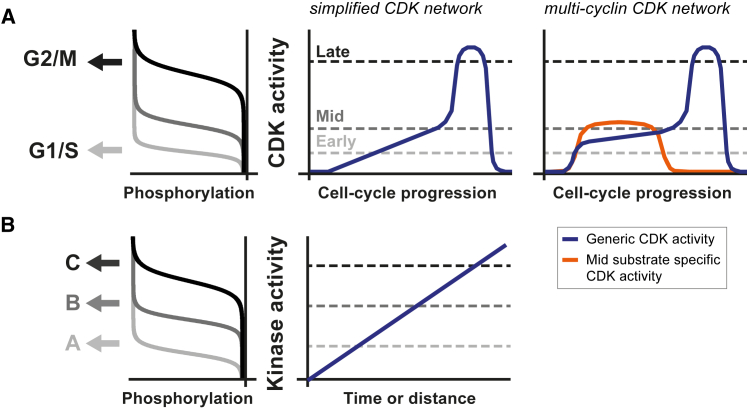
Summary Model (A) Simplified CDK network: a single CDK activity resolves substrates phosphorylation at distinct activity thresholds, resulting in the temporal ordering of downstream cell-cycle transitions. Multi-cyclin network: G1/S cyclins tune the profile of generic CDK activity and combine this with an enhanced specificity toward mid substrates. (B) Other kinase-regulated processes with multiple cellular outputs resolved in time or space could be organized by the same regulatory principle.

We have shown that clearly distinguished early and late patterns of substrate phosphorylation are observed when the cell cycle is driven by a single cyclin-CDK (Figure 2). The in vivo CDK-to-phosphatase activity ratio was measured at four points during the cell cycle and increased more than 10-fold between S phase and mitosis, while early substrates were found to be more sensitive to CDK activity than late substrates (i.e., become net phosphorylated at lower CDK activity levels) (Figures 3 and 4). Taken together, these data support a model where substrate phosphorylation is primarily ordered by CDK activity sequentially rising through distinct substrate-specific thresholds: early substrates become net phosphorylated first as CDK activity rises to a low level and then higher CDK activity levels phosphorylate late substrates at the end of the cell cycle, resulting in the orderly initiation of S phase and mitosis, respectively (Figure 7A). Further support for this model comes from our phosphoproteomics analysis of experiments where the order of S phase and mitosis is rearranged: these data are consistent with a direct causal relationship between CDK activity-dependent differential substrate phosphorylation and the order of cell-cycle events (Figures 5G and 5H). Furthermore, our data are consistent with the notion that the order of events within mitosis is also determined by a rising CDK activity surpassing sequential substrate-specific thresholds (Figure 4E) (Gavet and Pines, 2010a, Oikonomou and Cross, 2011).

### Combining Activity Thresholds and Cyclin-Substrate Specificity

Previous studies that have implicated activity thresholds have relied on certain genetic perturbations to test what *can* happen as opposed to what *does* happen (Coudreuse and Nurse, 2010, Fisher and Nurse, 1996, Gutiérrez-Escribano and Nurse, 2015). Our phosphoproteomics systems approach has allowed us to make a global assessment of the relative contributions made by activity thresholds and cyclin-specificity when multiple cyclin-CDK complexes are expressed in wild-type cells (Figure 6). This shows that G1/S cyclins combine cyclin-substrate specificity with activity thresholds to fine-tune the precise timings of substrate phosphorylation at the G1-to-S transition. Although well established in other systems, this is the first in vivo evidence for G1/S cyclin-substrate specificity in *S. pombe*. Despite this, a number of our observations indicate that activity thresholds play a more substantial role than cyclin-substrate specificity. First, essential S-phase-related proteins (e.g., Drc1, Sld3, Orc1, and Orc2) are robustly phosphorylated early in the cycle in cells run by a single cyclin-CDK. Second, when G1/S cyclin-CDKs are present, these early substrates are not subject to any obvious cyclin-substrate specificity; instead, G1/S cyclin-CDKs contribute to early substrate phosphorylation by providing additional generic CDK activity. Finally, when G1/S cyclins are present, we only observed substrates whose phosphorylation pattern was advanced from mid to early, never late to early. Therefore, where G1/S cyclin-substrate specificity does exist, it is always combined with an increased substrate sensitivity to generic CDK activity. It will be of interest to examine how and why some substrates are subject to G1/S cyclin specificity, while others are appropriately phosphorylated at G1/S by an elevated sensitivity to generic CDK activity alone.

These conclusions contrast with the generally accepted view that the major differences in the timing of CDK substrate phosphorylation are brought about by the biochemical specificity of different cyclin-CDK complexes toward sub-pools of certain substrates (Bhaduri and Pryciak, 2011, Brown et al., 1999, Kõivomägi et al., 2011, Loog and Morgan, 2005, Pagliuca et al., 2011, Peeper et al., 1993). Instead, we place greater emphasis on changes in generic CDK activity levels and propose that cyclin-substrate specificity provides a further layer of regulation to tune the timing of some substrates’ phosphorylation. Because our data show that activity thresholds and cyclin specificity operate concurrently, our conclusions are consistent with the substantial body of data in the literature concerning cyclin-substrate specificity, but come to a different assessment of their relative contribution.

Our work has used *S. pombe*, so it will be important to examine the role played by activity thresholds in other model eukaryotes. Clearly, the relative contribution made by cyclin specificity and quantitative changes in generic CDK activity may differ between systems, but the fact that there are important examples of cyclin-substrate specificity in other organisms does not preclude activity thresholds playing a substantial or even predominant role in those systems. Indeed, there are numerous reports of plasticity in the metazoan cell-cycle network, which genetically or biochemically demonstrate how certain cyclin-CDKs can fulfill the functions of other cyclin-CDK complexes (Uhlmann et al., 2011). This includes the major genetic redundancies among cyclins and CDKs in murine cells (Geng et al., 2003, Kalaszczynska et al., 2009, Kozar et al., 2004, Santamaría et al., 2007) and the observation that cyclin B1 has substantial S-phase-promoting activity in *Xenopus* egg extracts when it is synthetically relocalized to the nucleus (Moore et al., 2003). Such plasticity is consistent with, and readily accommodated by, the activity threshold model proposed here.

### Differential Substrate Sensitivity

What is the biochemical basis for the difference in substrate sensitivity? Phosphosites within the same protein tend to behave similarly, suggesting that the determinants for sensitivity function across an entire domain or protein, such as substrate docking interactions and/or the differential subcellular accessibility of substrates to cyclin-CDK (Figure 5C). In *S. pombe*, Cdc13-Cdc2 is predominantly nuclear throughout the cell cycle, but it concentrates at the SPB (spindle pole body; yeast centrosome equivalent) in late G2 and on the spindle during mitosis (Coudreuse and Nurse, 2010, Decottignies et al., 2001). Late substrates are enriched for SPB-localized and mitotic spindle-localized proteins, while early substrates are simply nuclear (Table S2) (Matsuyama et al., 2006). This suggests that the changing localization of cyclin-CDK may play a role in aiding late phosphorylation by altering substrate accessibility. However, this cannot explain the fact that early substrates are phosphorylated faster than late substrates, even in mitosis, especially given that there are many early and late substrates with similar localization patterns.

Instead, our experiments have shown that the putative cyclin-docking motifs (RxL motifs) in Sld3 and Mdb1 are required for their enhanced sensitivity to CDK activity (Figure 5E). This suggests that different substrate-specific thresholds are established by differences in affinity for cyclin-CDK. An important avenue of further research will be to systematically examine this in vitro, using purified proteins. Our preliminary observations predict that early substrates will have lower Km values than late substrates. The more finely resolved differences among late substrates could be accounted for by more subtle differences in their Kcat/Km values.

In vitro studies of substrate competition have demonstrated that the presence of higher-affinity CDK targets can inhibit CDK-mediated Wee1 phosphorylation and also enhance the non-linear response of Wee1 phosphorylation (Kim and Ferrell, 2007). One possibility is that the resolution between early and late phosphorylation, which we have described here, is aided by substrate competition in vivo. Our observation that the overexpression of early substrates causes a checkpoint-independent delay in the cell cycle is consistent with this possibility (Figure 5F).

### Rheostat-like Outputs in Kinase-Phosphatase Signaling

An important component to any such kinase-phosphatase signaling network is the phosphorylation turnover. Our measurements do not provide evidence for differences in phosphorylation turnover between early and late substrates (Figure 5B). They do, however, show that the turnover rates are remarkably fast for CDK substrates in general: the median phosphorylation half-life (2.2 min at 25°C) is less than 1% of the duration of a cell cycle. This rapid turnover suggests the presence of futile cycles of phosphorylation and dephosphorylation, which may be important for converting changes in CDK activity into stepwise threshold-like changes in net phosphorylation for any given substrate (Goldbeter and Koshland, 1981). Futile cycles could further assist the resolution between early and late thresholds by maintaining a small pool of dephosphorylated early substrates to suppress late phosphorylation via substrate competition. More generally, we suggest that other protein kinase-regulated processes with multiple cellular outputs resolved in time and/or space could be organized using the same regulatory principle, namely differential substrate sensitivity combined with rapid phosphatase-induced phosphorylation turnover, generating distinct substrate-specific activity thresholds (Figure 7B).

## STAR★Methods

### Key Resources Table

**Table:** 

REAGENT or RESOURCE	SOURCE	IDENTIFIER
**Antibodies**		

Rabbit polyclonal anti-Cdc13	Moreno et al., 1989	SP4
Rabbit polyclonal anti-Cdc2-Y15P	Cell Signaling Technologies	Cat#9111; RRID: AB_331460
Mouse monoclonal anti-alpha tubulin	Woods et al., 1989	TAT1
Rabbit polyclonal anti-Dis2	BioAcademia	Cat#63-119
Rabbit polyclonal anti-Dis2-T316P	BioAcademia	Cat#63-121
Mouse monoclonal anti-V5	AbD seroTEC	Cat#MCA1360; RRID: AB_322378
Rabbit polyclonal anti-HA	Cell Signaling Technologies	Cat#3724; RRID: AB_1549585
Mouse monoclonal anti-Flag	SIGMA	Cat#F3165; RRID: AB_259529
Horseradish peroxidase-conjugated goat anti-mouse	AbD SeroTEC	Cat#STAR120P; RRID: AB_567024
Horseradish peroxidase-conjugated donkey anti-rabbit	GE Healthcare	Cat#NA934; RRID: AB_772206

**Chemicals, Peptides, and Recombinant Proteins**		

Phos-tag acrylamide	Alpha Laboratories	Cat#AAL-107

**Critical Commercial Assays**		

Agilent 1200 HPLC	Agilent	Cat#G1312A
PolySULFOETHYL A column, 100 × 2.1 mm	PolyLC	Cat#102SE0502
Dionex UltiMate 3000 HPLC	Thermo Scientific	Cat#5041.0010
EASY-Spray C18 column, 75 μm × 50 cm	Thermo Scientific	Cat#ES803
Mass spectrometer: LTQ-Orbitrap Velos	Thermo Scientific	Cat#1239200
Mass spectrometer: LTQ-Orbitrap Velos Pro	Thermo Scientific	Cat#1239200

**Deposited Data**		

Raw mass spectrometry data	This paper	http://www.ebi.ac.uk/pride/archive/; PRIDE: PXD003598
Maxquant output files	This paper	http://www.ebi.ac.uk/pride/archive/; PRIDE: PXD003598
Maxquant output files after imputation and smoothing was applied	This paper	http://www.ebi.ac.uk/pride/archive/; PRIDE: PXD003598

**Experimental Models: Organisms/Strains**		

*S. pombe: cdc13-L-cdc2(as) Δ2 Δ13 ΔCCP SILAC+*	This paper	MS230 & MS213
*S. pombe: cdc13-L-cdc2(as) Δ2 Δ13 SILAC+*	This paper	MS122
*S. pombe: cdc13-L-cdc2AF(as) Δ2 Δ13 ΔCCP SILAC+*	This paper	MS86
*S. pombe: cdc13-L-cdc2(as) Δ2 Δ13 ΔCCP rad3Δ SILAC+*	This paper	MS87
*S. pombe: cdc2(as) SILAC+*	This paper	MS131
*S. pombe: cdc2(as) ΔCCP SILAC+*	This paper	MS200
*S. pombe: cdc2(as)*	This paper	MS278
*S. pombe: cdc2(as) ΔCCP*	This paper	MS282
*S. pombe: cdc2(as) ΔCCP rum1Δ*	This paper	MS67
*S. pombe: sld3-5flag orc2-v5 nsk1-GFP cdc13-L-cdc2(as) Δ2 Δ13 ΔCCP*	This paper	MS212
*S. pombe: sld3-5flag orc2-v5 nsk1-GFP cdc13-L-cdc2(as) Δ2 Δ13*	This paper	MS132
*S. pombe: cdc13-L-cdc2(as) Δ2 Δ13 ΔCCP*	P.N. lab collection	DC240 (MS108)
*S. pombe: cdc13-L-cdc2(as) Δ2 Δ13 ΔCCP nmt41-mdb1-v5*	This paper	MS306
*S. pombe: cdc13-L-cdc2(as) Δ2 Δ13 ΔCCP nmt41-mdb1(rxl:aaa)-v5*	This paper	MS307
*S. pombe: cdc13-L-cdc2(as) Δ2 Δ13 ΔCCP nmt41-drc1-v5*	This paper	MS308
*S. pombe: cdc13-L-cdc2(as) Δ2 Δ13 ΔCCP nmt41-drc1(rxl:aaa)-v5*	This paper	MS309
*S. pombe: cdc13-L-cdc2(as) Δ2 Δ13 ΔCCP nmt41-sld3-v5*	This paper	MS310
*S. pombe: cdc13-L-cdc2(as) Δ2 Δ13 ΔCCP nmt41-sld3(rxl:aaa)-v5*	This paper	MS311
*S. pombe: cdc13-L-cdc2(as) Δ2Δ13ΔCCP nmt1-*	This paper	MS312
*S. pombe: cdc13-L-cdc2(as) Δ2Δ13ΔCCP nmt1-drc1-v5*	This paper	MS313
*S. pombe: cdc13-L-cdc2(as) Δ2Δ13ΔCCP nmt1-sld3-v5*	This paper	MS314
*S. pombe: cdc13-L-cdc2(as) Δ2Δ13ΔCCP nmt1-sld3(rxl:aaa)-v5*	This paper	MS315
*S. pombe: cdc13-L-cdc2AF(as) Δ2Δ13ΔCCP nmt1-*	This paper	MS316
*S. pombe: cdc13-L-cdc2AF(as) Δ2Δ13ΔCCP nmt1-drc1-v5*	This paper	MS317
*S. pombe: cdc13-L-cdc2AF(as) Δ2Δ13ΔCCP nmt1-sld3-v5*	This paper	MS318
*S. pombe: cdc13-L-cdc2AF(as) Δ2Δ13ΔCCP nmt1-sld3(rxl:aaa)-v5*	This paper	MS319

**Recombinant DNA**		

Plasmid: Rip41x-drc1-v5	This paper	MSp108
Plasmid: Rip41x-drc1(rxl:aaa)-v5	This paper	MSp233
Plasmid: Rip41x-sld3-v5	This paper	MSp112
Plasmid: Rip41x-sld3(rxl:aaa)-v5	This paper	MSp234
Plasmid: Rip41x-mdb1-v5	This paper	MSp237
Plasmid: Rip41x-mdb1(rxl:aaa)-v5	This paper	MSp239
Plasmid: Rip3x	P.N. lab collection	ARC72 (MSp28)
Plasmid: Rip3x-drc1-v5	This paper	MSp102
Plasmid: Rip3x-sld3-v5	This paper	MSp106
Plasmid: Rip3x-sld3(rxl:aaa)-v5	This paper	MSp101

**Software and Algorithms**		

MaxQuant	Cox and Mann (2008)	http://www.coxdocs.org/doku.php?id=maxquant:start
Perseus	Tyanova et al. (2016)	http://www.coxdocs.org/doku.php?id=perseus:start
Prism	GraphPad Software	http://www.graphpad.com/

### Contact for Reagent and Resource Sharing

Further information and requests for reagents may be directed to and will be fulfilled by the Lead Contact, Matthew P. Swaffer (matthew.swaffer@crick.ac.uk).

### Experimental Model and Subject Details

#### *S. pombe* genetics and cell culture

*S. pombe* media and standard methods are as previously described (Moreno et al., 1991). Full genotypes of all strains used in this study are listed in Table S3. The following strains are previously reported: *cdc13-L-cdc2(as) Δ2 Δ13 ΔCCP, cdc13-L-cdc2AF(as) Δ2 Δ13 ΔCCP* (Coudreuse and Nurse, 2010), *ΔCCP* (Martín-Castellanos et al., 2000), *lys3-37 arg1-230 car2Δ* (Bicho et al., 2010), *sld3-5flag* (Fukuura et al., 2011), *rad3Δ* (Kim et al., 2010) and *cdc2(as)M17* (Aoi et al., 2014). Additional C-terminal tagging (*orc2-v5*), gene deletion (*rum1Δ*) and gene deletion marker switching was performed as previously described (Bähler et al., 1998). Derivatives of the above were constructed by crosses and confirmed by marker selection and/or colony PCR. Ectopic constructs for condition expression were constructed and integrated into the genome as follows. Commercially synthesized codon optimized DNA fragments encoding *drc1-V5*, *drc1(rxl:aaa)-v5*, *sld3-v5*, *sld3(rxl:aaa)-v5*, *mdb1-v5* or *mdb1(rxl:aaa)-v5* (IDT and Life Technologies) were sub-cloned downstream of the moderate strength nmt41 promoter in a Rip41x vector. NB the full RxL motif sequence is R/K-X-L-X_[0,1]_-F/Y/L/I/V/M/P and RxL:AAA constitutes the replacement of R/K-X-L with AAA. To generate Rip3x-*drc1-v5*, Rip3x-*sld3-v5* and Rip3x-*sld3(rxl:aaa)-v5* constructs the nmt41 promoter in the above Rip41x constructs was converted to the high strength nmt1 promoter using Q5 site directed mutagenesis (NEB). The above Rip3x and Rip41x constructs were PacI linearized and transformed to generate MS306-MS319. Stable leucine prototrophy was selected for and expression was confirmed by western blotting.

All experiments were performed in exponential growth. All experiments for proteomics were performed in SILAC adjusted media (EMM (6 mM ammonium chloride) + 0.25 mg/ml leucine, 0.15 mg/ml uridine, 0.04 mg/ml arginine and 0.03 mg/ml lysine) (Bicho et al., 2010), unless stated otherwise. For heavy labeled samples, cells were first grown for > 8 generations in SILAC media supplemented with heavy arginine (L-arginine:HCL (U13C6, 99%)) and heavy lysine (L-lysine:2HCL (U13C6, 99%)) isotopes (Cambridge Isotope Laboratories Inc.). All other experiments were performed in EMM4S except where the use of YE4S media is specified (Moreno et al., 1991). G2 arrests were imposed by treatment with 0.5-1 μM 1-NmPP1 for approximately one generation (EMM4S & SILAC media: 180 min at 32°C & 5 hr at 25°C, YE4S media: 130 min at 32°C). Time (min) after wash & release is with respect to the initial re-suspension of cells during the first of three washes, unless stated otherwise.

Cell-cycle progression was monitored by DNA content and cell & nuclear division. DNA content was determined by flow cytometry (FACS): ethanol (70%, v/v) fixed cells were washed and resuspended in 50 mM sodium citrate before incubation with RNase A (0.1 mg/ml) (SIGMA) (37°C, overnight). DNA was then stained with propidium iodide (2 μg/ml) (SIGMA) before sample sonication and DNA content per event was acquired for 10,000 events on a BD LSR-Fortessa. DNA content is displayed on a log scale after gating for single, whole cells in FlowJo X (Treestar Inc). Nuclear division and cell division were scored in heat fixed samples by monitoring DNA (DAPI, SIGMA) and septum formation (calcofluor, SIGMA), respectively. Cell size at division was measured from live septated (calcofluor stained, SIGMA) cells. Samples were imaged on a Zeiss Axioskop microscope, 63 × /1.4 NA objective and cell photos were taken with a QICAM Fast Digital Camera.

### Method Details

#### Protein extractions and western blotting

Protein samples were taken as follows. Cell cultures were quenched by adding 100% (w/v) ice cold trichloroacetic acid (TCA) to a final concentration of 10%. Cells were kept on ice for > 20 min, pelleted and washed in −20°C acetone. Cell pellets were then washed and resuspended (100-200 μl) in lysis buffer (8 M urea + 50 mM ammonium bicarbonate + cOmplete Mini EDTA-free protease inhibitor cocktail (Roche) + PhosSTOP phosphatase inhibitor cocktail (Roche)). 1.2 ml acid washed glass beads (0.4 mm, SIGMA) were added to cell suspensions, which were then beaten to break cells (FastPrep120). Cell debris was pelleted (14,000 rpm, 5 min) and the supernatant was recovered as a protein sample (stored at −80°C). Protein samples for Lambda phosphatase (NEB) treatment were extracted as above, with the omission of PhosSTOP.

Phosphorylation dependent mobility shifts for Orc2, Sld3, Bir1 and Mdb1 were resolved using Phos-tag (Alpha Laboratories) supplemented SDS-PAGE under neutral pH conditions (Kinoshita and Kinoshita-Kikuta, 2011). Protein detection by western blotting was performed using the following antibodies. Cdc13-L-Cdc2: 1:6,000 SP4 antibody (rabbit polyclonal) (Moreno et al., 1989). Cdc2-Y15P: 1:500 anti-Cdc2-Y15P (rabbit polyclonal) (#9111, Cell Signaling Technologies). Alpha tubulin: 1:10,000 TAT1 (mouse monoclonal) (Woods et al., 1989). Bir1: 1:1,000 anti-Bir1 (rabbit polyclonal) (Tsukahara et al., 2010). Dis2: 1:1,000 anti-Dis2 (rabbit polyclonal) (63-119, BioAcademia). Dis2-T316P: 1:1,000 anti-Dis2-T316P (rabbit polyclonal) (63-121, BioAcademia). V5 epitope tag: 1:1,000 SV5-PK1 (mouse monoclonal) (MCA1360, AbD seroTEC). HA epitope tag: 1:1,000 anti-HA C29F4 (rabbit polyclonal) (#3724, Cell Signaling Technologies). Flag epitope tag: 1:1,000 anti-flag M2 (mouse monoclonal) (F3165, SIGMA). Secondary antibodies: 1:25,000 horseradish peroxidase-conjugated donkey anti-rabbit (NA934, GE Healthcare) or goat anti-mouse (STAR120P, AbD SeroTEC). Signal was detected using Amersham ECL Prime Western Blotting Detection Reagent (GE Healthcare) or SuperSignal West Femto Maximum Sensitivity Substrate (34095, Life Technologies) and imaged on an ImageQuant LAS 4000.

#### Sample preparation for mass spectrometry

Heavy labeled or light labeled protein samples were mixed with a light labeled or heavy labeled reference protein sample respectively. Protein samples were mixed in a 1:1 protein amount ratio (protein concentration determined by Bradford assay (Bio-Rad)). 1mg of protein sample (after mixing) was reduced with 5mM dithiothreitol (DTT) (56°C, 25 min), alkylated with 10 mM iodoacetamide (room temperature, 30 min, dark) and quenched with 7.5 M DTT. Samples were then diluted with 50 mM ammonium bicarbonate to reduce the urea concentration to < 2 M, prior to trypsin digestion (37°C, overnight). Peptides were acidified to 0.4% trifluoroacetic acid (TFA) and centrifuged (14,000 rpm, 4°C, 30 min). Peptides were then desalted using a C_18_ SepPak Lite (130 mg bed volume) under vacuum and dried. To ensure complete digestion, peptides were further digested using Lys-C in 10% acetonitrile, 50 mM ammonium bicarbonate (37°C, 2 h), followed by trypsin digestion (37°C, overnight). Digested peptides were then desalted again and dried.

Phosphopeptide enrichment using titanium dioxide (TiO_2_) was carried out as follows. Dried peptide mixtures were re-suspended in 1 M glycolic acid + 80% acetonitrile + 5% trifluoroacetic acid, sonicated (10 min) and added to titanium dioxide beads (5:1 (w/w) beads:protein). The beads were washed using 80% acetonitrile + 1% trifluoroacetic acid followed by 10% acetonitrile + 0.2% trifluoroacetic acid, and dried under vacuum centrifugation. Flow-through fractions were retained for analysis of non-phosphorylated peptides. Phosphopeptides were eluted from the beads by adding 1% ammonium hydroxide followed by 5% ammonium hydroxide, and dried by vacuum centrifugation. Dried phosphopeptides were re-suspended in 100 μl of 1% trifluoroacetic acid and sonicated (15 min). A C_18_ membrane was packed into a 200 μl pipette tip and washed using methanol and equilibrated with 1% trifluoroacetic acid. The peptides were loaded onto the Stage Tip and washed with 1% trifluoroacetic acid followed by elution with 80% acetonitrile + 5% trifluoroacetic acid. The eluted peptides were again dried under vacuum centrifugation. For analysis of non-phosphorylated peptides, the stored flow-through fractions were dried, desalted using a C_18_ SepPak Lite (130 mg bed volume) under vacuum and dried. Non-phosphorylated peptides were separated into 12 fractions using strong cation exchange (SCX) liquid chromatography (PolySULFOETHYL A column, 100 × 2.1 mm, 5 μm, 200 A) and dried to 250 μl.

An LTQ-Orbitrap Velos was used for data acquisition of phosphopeptides and an LTQ-Orbitrap Velos Pro was used for data acquisition of non-phosphorylated peptides. Both instruments were coupled to UltiMate 3000 HPLC systems for on-line liquid chromatographic separation. Phosphopeptide mixtures were re-suspended in 35 μl 0.1% trifluoroacetic acid and injected three times (10 μl per injection). Each run consisted of a 3 hr gradient elution (75 μm × 50 cm C_18_ column) with one activation method per run: Collision Induced Dissociation (CID), Multi-Stage Activation (MSA) and Higher energy collision Dissociation (HCD). Non-phosphopeptide mixtures were diluted 1:10 (v/v) in 0.1% trifluoroacetic acid and injected three times (10 μl per injection). Each run consisted of a 3 hr gradient elution (75 μm × 50 cm C_18_ column) with CID used as the activation method.

#### Processing and analysis of mass spectrometry data

MaxQuant (version 1.3.0.5) was used for all data processing. The data was searched against a UniProt extracted *S. pombe* proteome fasta file amended to included common contaminants and account for the altered genetic background of the reference sample (P0000 = Cdc13-L-Cdc2). A decoy database containing reverse sequences was used to estimate false discovery rates and set the false discovery rate at 1%. Default MaxQuant parameters were used with the following adjustments: Phospho(STY) was added as a variable modification (for the phospho-samples only), Lys6 and Arg6 were the heavy labels, ‘Filter labeled amino acids’ was deselected, re-quantify was selected with the instruction to keep low-scoring versions of identified peptides within parameter groups and match between runs was selected. Experiment and sample codes used to name MaxQuant output files are listed in Table S4. The experimental groups in which raw data was searched in MaxQuant are listed in Table S4. An overview of the number of sites or proteins quantified in each experiment is provided in Table S5.

MaxQuant output files were imported into Perseus (version 1.4.0.2) and the normalized heavy-to-light (H:L) ratios were used for all subsequent analysis. For phosphopeptides, only phosphosites with > 0 valid values quantified and a localization probability > 0.9 were used for subsequent data analysis, after removal of reverse and contaminant peptides. Data imputation and smoothing was performed in R. All linear regressions, non-linear curve fitting (sigmoidal function and exponential decay), interpolation, AUC (Area Under Curve) calculations, Spline calculation and Mann-Whitney U tests were performed in Prism 6 on data exported from Perseus.

### Quantification and Statistical Analysis

#### Defining CDK substrates and calculating phosphorylation half-lives and dephosphorylation rates

The relative phosphorylation of phosphosites after CDK inactivation in S phase and mitosis was used to define CDK substrates. The CCC5977 (S phase) and CCC5978 (mitosis) datasets were filtered for phosphorylation events at S/T-P (minimal CDK consensus site). The following four criteria were the used to define CDK substrate sites (Figure S1G). *i)* Given M00 is a comparison between biological replicates (i.e., heavy and light samples are equivalent) the CCC5978 dataset was filtered to only include phosphosites for which 2 > H:L (M00) > 0. 5 (i.e., deviated less than twofold from the theoretical expected value (H:L(M00) = 1)). CCC5977 was filtered to only include phosphosites identified in S00 for which H:L(S00) > 0.25. *ii)* Phosphosites that had ratios measured in less than four out of seven time points were excluded. *iii)* Sites that had a H:L ratio at 1, 3, 6, 9 and/or 12 min after CDK inactivation less than half the value of the initial time point (0 min) were kept. *iv)* A non-linear one-phase exponential decay was fitted to the data using the least-squares (ordinary) fitting method and was used to calculate phosphorylation half-life and dephosphorylation rate values (Prism 6) (related to Figures 1, 5A, 5B, S3K, and S3L). The curve was constrained such that K > 0 and Plateau > 0. CDK substrates were defined as sites that fitted an exponential decay with R squared > 0.9 and a plateau < 0.5. Sites that did not pass the above criteria were screened for anomalous ratio measurements as follows. Each time point (except 0 min) was independently removed from the dataset. Each modified dataset was re-assessed by the four above criteria (*i-iv*), and if a site was now defined as a substrate it was included as a substrate with the respective anomalous value removed before calculation of the phosphorylation half-life and dephosphorylation rate. Where the removal of multiple values independently permitted a site to pass the four above criteria, the one that gave the highest one-phase exponential decay R squared value was used. Phosphosites defined as CDK substrates and the corresponding half-life and dephosphorylation rate values are listed in Table S1.

For the comparison of half-lives between biological repeats (Figure 1B), half-life values were re-calculated from both experiments using H:L ratios up to and including 12 min after CDK inactivation in mitosis, because samples were only analyzed for the biological repeat between 0 and 12 min after CDK inactivation. Half-lives were only calculated for sites that conformed to criteria *i-iv* in both experiments. NB anomaly screening was not performed for this analysis.

#### Consensus sequences and annotation enrichments

The amino acid distribution surrounding a set of phosphosites was generated in Perseus using regular expressions. Annotation enrichment was performed in Perseus. Gene ontology annotation lists are default settings in Perseus. Additional annotation lists were generated using the curated *S. pombe* protein ortholog list (Wood et al., 2012), *S. cerevisiae* Cdc28 substrate list (Holt et al., 2009), human Cdk1 & Cdk2 substrate list (Hornbeck et al., 2015) and the proteome-wide microscopy based *S. pombe* protein localization database (Matsuyama et al., 2006). Enrichment analysis of CDK substrate sites was performed against all phosphosites in CCC5977&CCC5978 by Fisher exact test (B-H FDR < 0.02). Enrichment of early, mid and late substrate sites was performed against all phosphosites in CCC6254 (after imputation, see below) by Fisher exact test (B-H FDR < 0.02).

#### Imputation and smoothing

Imputation was applied to replace missing values. Phosphosites or protein groups with at least 50% valid values quantified within the respective dataset were processed for imputation. Missing values were imputed using the R package DMwR (Torgo, 2010). Default parameters of knnImputation function were used. The number of sites or proteins to which imputation was applied and the number of imputed values in any given dataset is listed in Table S5. Less than 15% of any dataset constituted imputed values after imputation. For data smoothing an R script was applied in which the five nearest neighbors for each site were identified by Euclidian distance. The mean ratio of the five nearest neighbors’ ratios was then substituted for each original ratio. Imputed and smoothed data is used only where stated.

#### Hierarchical clustering analysis

Hierarchical clustering was performed in Perseus (Settings: cluster rows, Euclidian distance, do not presuppose K-means) on datasets with missing values imputed (sees above). Related to Figures 1C and 2C.

#### Calculation of CDK substrate phosphorylation rates

CDK substrate phosphorylation rates were quantified as follows. Substrate sites that were dephosphorylated after transient CDK inactivation (i.e., L:H < 0.5 at 0 min from CDK restoration (before 1-NmPP1 washout)) were analyzed to calculate phosphorylation rates and the respective median relative phosphorylation values for early, mid and late substrates. A linear regression line was fitted (Prism 6) to values during the period of approximately linear increase in relative phosphorylation for individual substrate sites, and the median relative phosphorylation of early, mid and late substrate sites. Relative rate measurements were derived from the slope of the linear regression line. Related to Figures 3 and S3A–S3D.

#### Calculation of CDK substrate phosphorylation 1-NmPP1 IC_50_ and Hill slope values

The relative phosphorylation of CDK substrate sites across a range of 1-NmPP1 concentrations was fitted to a non-linear sigmoidal curve (four-parameter logistic function) using the least-squares (ordinary) fitting method (Prism 6). The curve was constrained such that the Hill slope < 0 and Bottom > 0. Outlier detection was used (Q = 10%). Phosphosites with fitted curves (R square > 0.9, Bottom < 0.5 and Top/Bottom > 2) were used to derive IC_50_ and Hill slope values. IC_50_ and Hill slope values for CDK substrate sites are listed in Table S1. Related to Figures 4 and S3E–S3K.

#### Calculation of the phosphorylation across G2/M AUC value

The total relative phosphorylation (i.e., the integral of phosphorylation (Area Under Curve, AUC)) was calculated (Prism 6) as a proxy for the timing and extent of a site’s phosphorylation during progression from early G2 through to mitosis (50-100 min after release). AUC values were calculated from data after imputation to replace missing values and data smoothing. The peak in binucleation during this mitosis is < 50% synchronous (Figure S1B) meaning that sites that are phosphorylated earlier in G2/M will be phosphorylated in a larger proportion of cells in the culture at any one time as compared to sites phosphorylated later in mitosis and therefore will have a larger peak in phosphorylation and a larger AUC. AUC values for CDK substrate sites are listed in Table S1. Related to Figure 4E.

#### Calculation of in vivo G1/S cyclin specificity score

The extent of cyclin-substrate specificity was assessed by calculating an in vivo G1/S cyclin specificity score for early and mid substrates as follows. Sigmoidal curves fitted to the median relative phosphorylation values of early and mid substrates across a range of 1-NmPP1 concentrations (Figure S3E) were used to interpolate 1-NmPP1 concentrations for the median relative phosphorylation values for early and mid substrates in Figures 6C and 6D (Prism 6). These values were then normalized to the interpolated 1-NmPP1 concentrations for the median relative phosphorylation of late substrates. Normalized values were then inverted and a ratio for *wt / ΔCCP* or *ΔCCP rum1Δ / ΔCCP* was calculated to give to give a semiquantitative estimate of G1/S cyclin specificity in vivo (A.U.). Substrates subject to G1/S cyclin specificity will give an in vivo specificity score > 1 in *wt / ΔCCP* and ∼1 in *ΔCCP rum1Δ / ΔCCP*. Related to Figures 6F and 6G.

### Data and Software Availability

Mass spectrometry proteomics data have been deposited to the ProteomeXchange Consortium via the PRIDE partner repository (http://www.ebi.ac.uk/pride/archive/) under the dataset identifier PRIDE: PXD003598. Raw data, all MaxQuant output files and MaxQuant output Phospho (STY)Sites.txt and ProteinGroups.txt files after imputation and smoothing have been deposited. File names and the corresponding experimental samples are listed in Table S4.

## Author Contributions

M.P.S., A.P.S., and P.N. conceived the experiments. M.P.S. designed and performed the experiments. A.W.J. and H.R.F. performed mass spectrometry and processed raw data. M.P.S. analyzed processed data. M.P.S. and P.N. wrote the manuscript.

## Supplemental Information

Table S1. CDK Substrate Sites, Related to Figures 1, 2, 4, 5A, and 5B List of all phosphosites defined as CDK substrate sites, early, mid or late annotation and the phosphorylation half-life, dephosphorylation rate, 1-NmPP1 IC_50_, 1-NmPP1 Hill Slope and area under curve (AUC) values calculated for each site. See STAR Methods for details of how CDK substrate sites were defined and how the corresponding values were calculated.

Table S2. Annotation Enrichment of CDK Substrate Sites, Related to Figures 1 and 2 GO and other annotation categories for which early, mid, late or all CDK substrate sites are significantly and >1.5 fold enriched. See STAR Methods for details of how the enrichment analysis was performed.

Table S3. *S. pombe* Strain Genotypes, Related to STAR Methods

Table S4. SILAC Experiments and Experiment Codes, Related to STAR Methods List of all protein samples processed (n=146) for phosphoproteomics and/or proteomics analysis. Raw data file names for each protein sample are listed. Sample ID and experiment codes used to name the MaxQuant output files are listed. Green rows separate phosphoproteomic samples whose raw data files were independently searched in MaxQuant.

Table S5. Overview of SILAC Phosphoproteomics and Proteomics Data and Imputation, Related to STAR Methods List of SILAC-based each experiment with the total number of phosphosites or proteins quantified per experiment. The number of phosphosites or proteins processed for imputation and the number of values imputed in any given dataset are listed. See Table S4 for details of experimental codes.
